# Exosomes of endothelial progenitor cells repair injured vascular endothelial cells through the Bcl2/Bax/Caspase-3 pathway

**DOI:** 10.1038/s41598-024-55100-x

**Published:** 2024-02-23

**Authors:** Wei Tan, Yanling Li, Lu Ma, Xinying Fu, Qingyin Long, Fanchen Yan, Wanyu Li, Xiaodan Liu, Huang Ding, Yang Wang, Wei Zhang

**Affiliations:** 1 College of Integrated Chinese and Western Medicine, Key Laboratory of Hunan Provincial for Integrated Traditional Chinese and Western Medicine on Prevention and Treatment of Cardio-Cerebral Diseases, Hunan University of Chinese Medicine, Hunan, 410208 China; 2 Institute of Integrative Medicine, Key Laboratory of Hunan Province for Liver Manifestation of Traditional Chinese Medicine, Xiangya Hospital, Central South University, Hunan, 410008 China

**Keywords:** Exosomes of endothelial progenitor, Carotid artery injury, Apoptosis, Bcl2/Bax/Caspase-3 pathway, Stem cells, Molecular biology

## Abstract

The main objective of this study is to evaluate the influence of exosomes derived from endothelial progenitor cells (EPC-Exo) on neointimal formation induced by balloon injury in rats. Furthermore, the study aims to investigate the potential of EPC-Exo to promote proliferation, migration, and anti-apoptotic effects of vascular endothelial cells (VECs) in vitro. The underlying mechanisms responsible for these observed effects will also be thoroughly explored and analyzed. Endothelial progenitor cells (EPCs) was isolated aseptically from Sprague–Dawley (SD) rats and cultured in complete medium. The cells were then identified using immunofluorescence and flow cytometry. The EPC-Exo were isolated and confirmed the identities by western-blot, transmission electron microscope, and nanoparticle analysis. The effects of EPC-Exo on the rat carotid artery balloon injury (BI) were detected by hematoxylin and eosin (H&E) staining, ELISA, immunohistochemistry, immunofluorescence, western-blot and qPCR. LPS was used to establish an oxidative damage model of VECs. The mechanism of EPC-Exo repairing injured vascular endothelial cells was detected by measuring the proliferation, migration, and tube function of VECs, actin cytoskeleton staining, TUNEL staining, immunofluorescence, western-blot and qPCR. In vivo, EPC-Exo exhibit inhibitory effects on neointima formation following carotid artery injury and reduce the levels of inflammatory factors, including TNF-α and IL-6. Additionally, EPC-Exo downregulate the expression of adhesion molecules on the injured vascular wall. Notably, EPC-Exo can adhere to the injured vascular area, promoting enhanced endothelial function and inhibiting vascular endothelial hyperplasia Moreover, they regulate the expression of proteins and genes associated with apoptosis, including B-cell lymphoma-2 (Bcl2), Bcl2-associated x (Bax), and Caspase-3. In vitro, experiments further confirmed that EPC-Exo treatment significantly enhances the proliferation, migration, and tube formation of VECs. Furthermore, EPC-Exo effectively attenuate lipopolysaccharides (LPS)-induced apoptosis of VECs and regulate the Bcl2/Bax/Caspase-3 signaling pathway. This study demonstrates that exosomes derived from EPCs have the ability to inhibit excessive carotid intimal hyperplasia after BI, promote the repair of endothelial cells in the area of intimal injury, and enhance endothelial function. The underlying mechanism involves the suppression of inflammation and anti-apoptotic effects. The fundamental mechanism for this anti-apoptotic effect involves the regulation of the Bcl2/Bax/Caspase-3 signaling pathway.

## Introduction

Cardiovascular Diseases (CVDs) remain the leading cause of mortality worldwide^1^. Percutaneous coronary intervention (PCI) and stenting are commonly used as the primary procedures for the treatment of CVDs, such as myocardial infarction^2^. However, these interventions have been associated with several complications, including local thrombosis and vascular narrowing induced by excessive vascular endothelial hyperplasia^3,4^. Endothelial barrier integrity is necessary to maintain vascular homeostasis and avoid thrombosis^5^. Some implications of a compromised endothelial barrier function include destroyed endothelial junctions, increased vascular permeability, and local micro-thrombosis^6,7^. Following endothelial damage, a multitude of local injury tissue cytokines is generated^8^, stimulating inflammation and apoptosis-related proteins, thereby triggering apoptosis. Consequently, inhibiting vascular endothelial hyperplasia, and preventing injury-induced endothelial cell death to maintain endothelial function, is excellent avenues for inhibiting vascular re-narrowing.

According to recent research^9,10^, the impact of paracrine factors on endothelial progenitor cells (EPCs), a type of adult stem cell, is essential in disease treatment. Stem cell therapy, such as EPCs, has emerged as a potential treatment approach for CVDs^11^. There is convincing evidence that EPCs, as a regenerative cargo, are recruited to areas of vascular injury to provide VECs for neointimal growth and to stimulate the secretion of growth factors by neighboring cells^12^. However, due to potential immune rejection, chromosomal abnormalities, and emboli formation, only a few EPCs survive after transplantation. The paracrine signaling of EPCs is suggested to play an important role in the repair of vascular injury^13^. Exosomes (Exos) are crucial cell-secreted paracrine factors abundant in lipids, proteins, and nucleic acids^14,15^. Exos have been associated with a higher survival and transplantation compared to stem cells in circulation, as well as a higher efficacy in CVDs prevention and treatment, particularly by angiogenesis and decreasing arterial remodeling^16^. Moreover, exosomes derived from endothelial progenitor cells (EPC-Exo) may exert a protective effect on endothelial cells by promoting their proliferation and migration, thereby enhancing the internal endothelial barrier function by facilitating the retention of healthy endothelial cells^17^. The mechanism underlying this process may be associated with reduced cell death and improved cell survival.

This study aimed to explore whether EPC-Exo administration could inhibit neointimal hyperplasia in a rat carotid artery balloon injury (BI) model, promote the repair of endothelial cells in intimal injury, and enhance endothelial function. Moreover, we investigated whether EPC-Exo treatment has effects on inhibiting inflammation and regulating apoptosis. Furthermore, we explored the Bcl2/Bax/Caspase-3 pathway to elucidate the molecular mechanisms underlying EPC-Exo activity.

## Result

### EPCs identification and characterization of EPC-Exo

**Figure 1:**
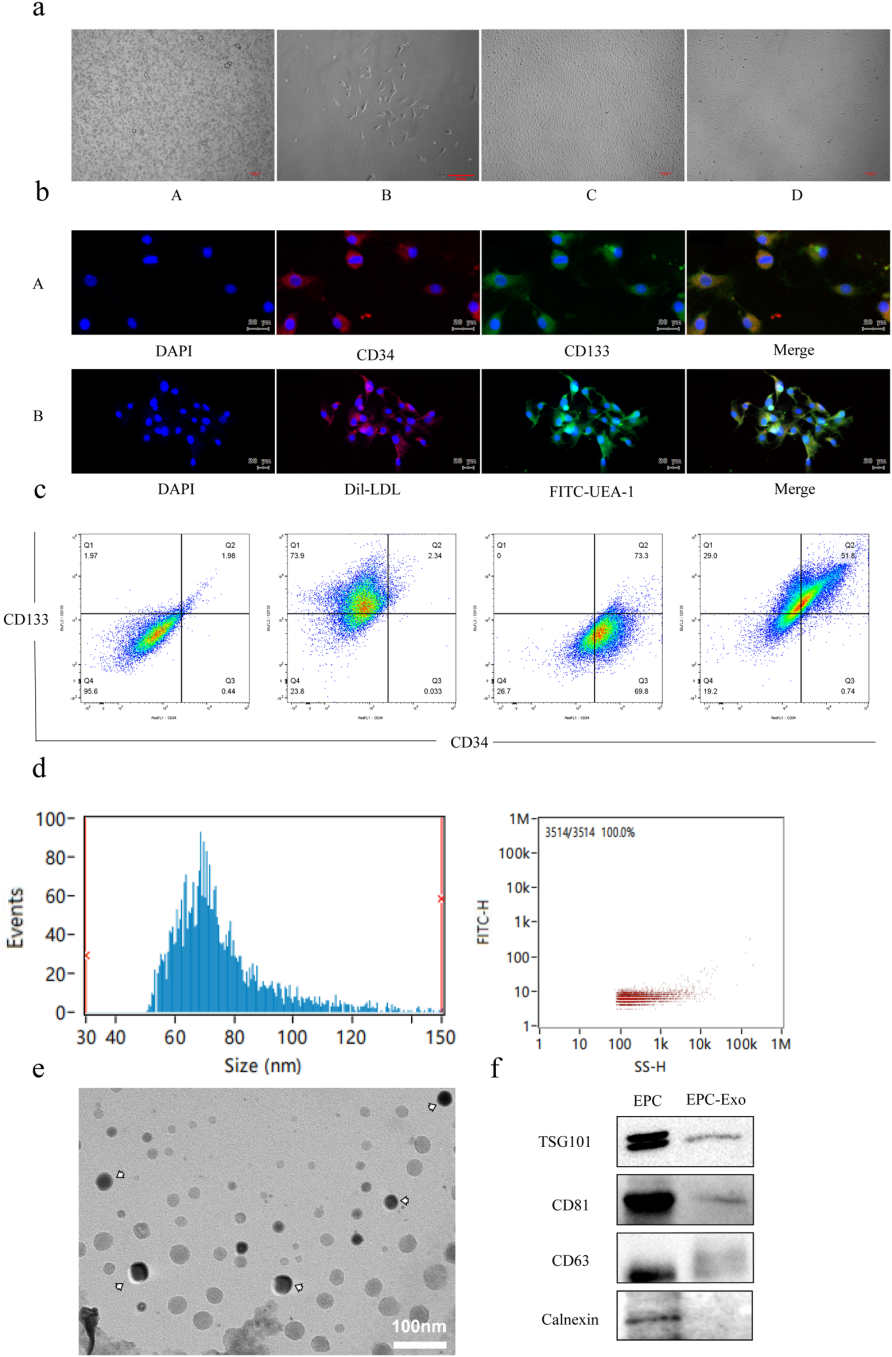
Characteristics and functional validation of EPCs and EPC-Exo. (**a**) Growth morphology of EPCs A: 0 days cell form is a transparent circular shape. B: EPCs grow into long shuttle cells in 7 days (scale bar = 50 μm). C: EPCs growth resembles a paved stone. D: The shape of EPCs in P2 generation (scale bar = 100 μm). (**b**) Fluorescent identification of EPCs. A: Specific marker identification of cells. B: Pochism function appraisal of cells. EPCs appear as double-positive and can be recognized as such (scale bar = 20 μm). (**c**) Flow cytometry of EPCs. (**d**) Particle size analysis and particle concentration of EPC-Exo. (**e**) Cup-shaped morphology of EPC-Exo (arrowhead) assessed by transmission electron microscope (scale bar = 20 μm). (**f**) Representative images of western blot showing the exosomes protein markers.

Initially, On the first day, EPCs isolated from the bone marrow appeared as brilliant, round dots. In the 48–72 h cultivation period, the cells transitioned to a substrate-attached growth, accompanied by alterations in cellular morphology. Subsequently, EPCs exhibited a spindle-like morphology on day 7 of culture. After 14 days of cell culture, colonies of EPCs appeared within the flask, gradually assuming a cobblestone-like morphology. After passage cultivation, we observed a uniform distribution of cells with consistent growth (Fig. 1a). Immunocytochemistry analysis revealed that EPCs express two specific markers: CD133 and CD34. At the same time, immunostaining confirmed that EPCs could take up Human Dil-labeled acetylated low-density lipoprotein (Dil-LDL) and integrate with specific markers such as FITC labeled ulex europaeus agglutinin 1 (FITC-UEA-1) (Fig. 1b). The flow cytometry assay results suggested that the P2-generation EPCs expressed specific markers including CD34 (73.8%) and CD133 (58%), with a double-positive expression accounting for 52.2% of the total (Fig. 1c). These data indicated that the cells used in our experiments are EPCs. Exosome particle size analysis revealed that the exosome diameters ranged between 30 and 150 nm, with an average of 75.29 nm. Most exosome diameters (90.35%) conformed with the standard exosome size. Exosome particle concentration was 1.32 × 10^10^ particles/ml (Fig. 1d). Consistent with the typical exosome, transmission electron microscopy revealed that exosomes were "cup-shaped" and complete (Fig. 1e). Additionally, WB analysis revealed that exosomes were positive for exosome-specific markers, including tumor susceptibility gene 101 protein (TSG101), CD81, and CD63 (Fig. 1f-Supplementary material Figure S1). These data confirmed the identification and characterization of exosomes.

### EPC-Exo could attach to the wall of a damaged carotid artery, improve endothelial function, and inhibit neointimal hyperplasia in rat carotid artery after balloon injury

**Figure 2:**
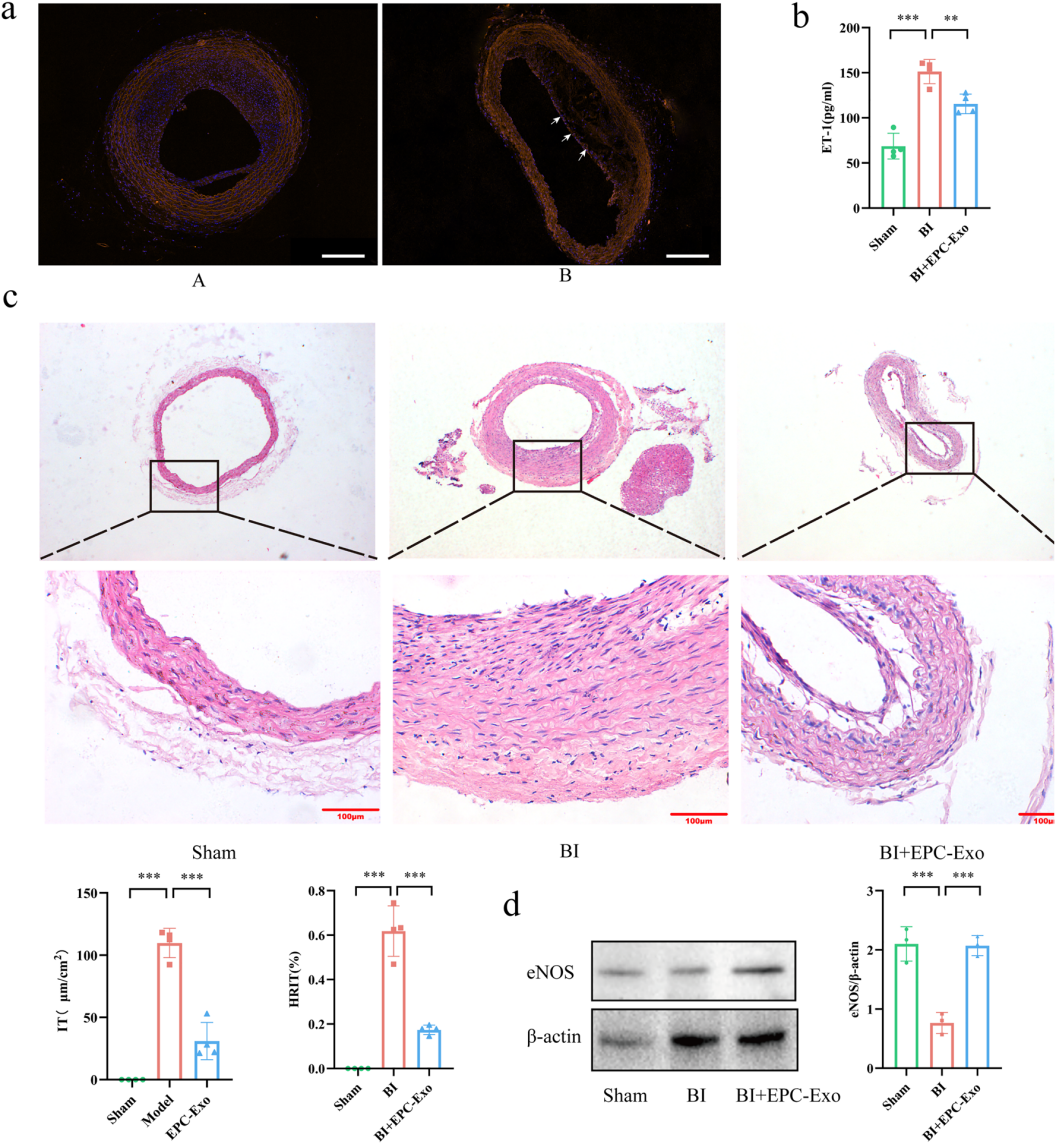
EPC-Exo could adhere to the wall of a damaged carotid artery, improve endothelial function and inhibited neointimal hyperplasia in rat carotid artery after balloon injury. (**a**) PKH26-labelled EPC-Exo with orange fluorescence in injured endothelial was monitored on Day 7 after injury (arrowhead). On the contrary, the BI model group did not monitor fluorescence compared with EPC-Exo treatment. A: BI model on Day 7. B: BI + PKH26-labelled EPC-Exo on Day 7 (scale bar = 200 μm). (**b**) Quantitative level of endothelin-1 (ET-1) in serum. EPC-Exo could decrease expression of ET-1 in serum. (**c**) In HE staining, the endothelial hyperplasia and the quantitative data of its thickness were observed. EPC-Exo therapy substantially lowers endothelial hyperplasia as well as IT and HRIT values (scale bar = 100 μm). (**d**) Representative western blot images and quantitative data of eNOS expression in carotid artery are shown. β-actin served as the reference protein (***p < 0.001, **p < 0.01, *p < 0.05, n ≥ 3 per group). The data are displayed as the M ± SD.

We injected phosphate buffer saline (PBS) and EPC-Exo into the rats after injury to assess attachment to the injured luminal surface in vivo. The exosome group animals were injected with PKH26-labeled EPC-Exo (Exo-PKH26). The distribution of Exo-PKH26 with orange fluorescence in injured carotid arteries was monitored on day 7 after injury. Compared to the balloon injury (BI) model group, Exo-PKH26 was detected in the vascular endothelium with less neointimal hyperplasia. The results showed that EPC-Exo could attach to the wall of a damaged carotid artery (Fig. 2a). Vascular morphology was studied through hematoxylin and eosin (H&E) staining to investigate the effects of EPC-Exo in the arterial wall after BI. The carotid intima in the sham group was in good condition and with no increase in neointimal thickness. However, compared to the sham group, the carotid intima in the BI model group was significantly thickened with a narrower lumen area. Endothelin-1 (ET-1) is a VECs-synthesized polypeptide, which could induce vascular contraction and increase single-core cell adhesion^18^. The serum ELISA results revealed elevated levels of ET-1 in the BI model group, which were subsequently reversed by the injection of EPC-Exo (Fig. 2b). Furthermore, the H&E results revealed that compared to the sham group, intimal thickness (IT) and hyperplasia ratio of intima thickness (HRIT) substantially increased in the BI model group, but these effects were alleviated by EPC-Exo treatment (Fig. 2c). At the same time, EPC-Exo treatment upregulated endothelial nitric oxide synthases (eNOS), which were analyzed to evaluate endothelial function (Fig. 2d-Supplementary material Figure S2). Overall, these data showed that EPC-Exo could attach to the wall of damaged carotid arteries, thereby improving endothelial function and inhibiting neointimal hyperplasia in rat after carotid artery after BI.

### EPC-Exo alleviated inflammation and regulated the expression of apoptosis-related genes and proteins

**Figure 3:**
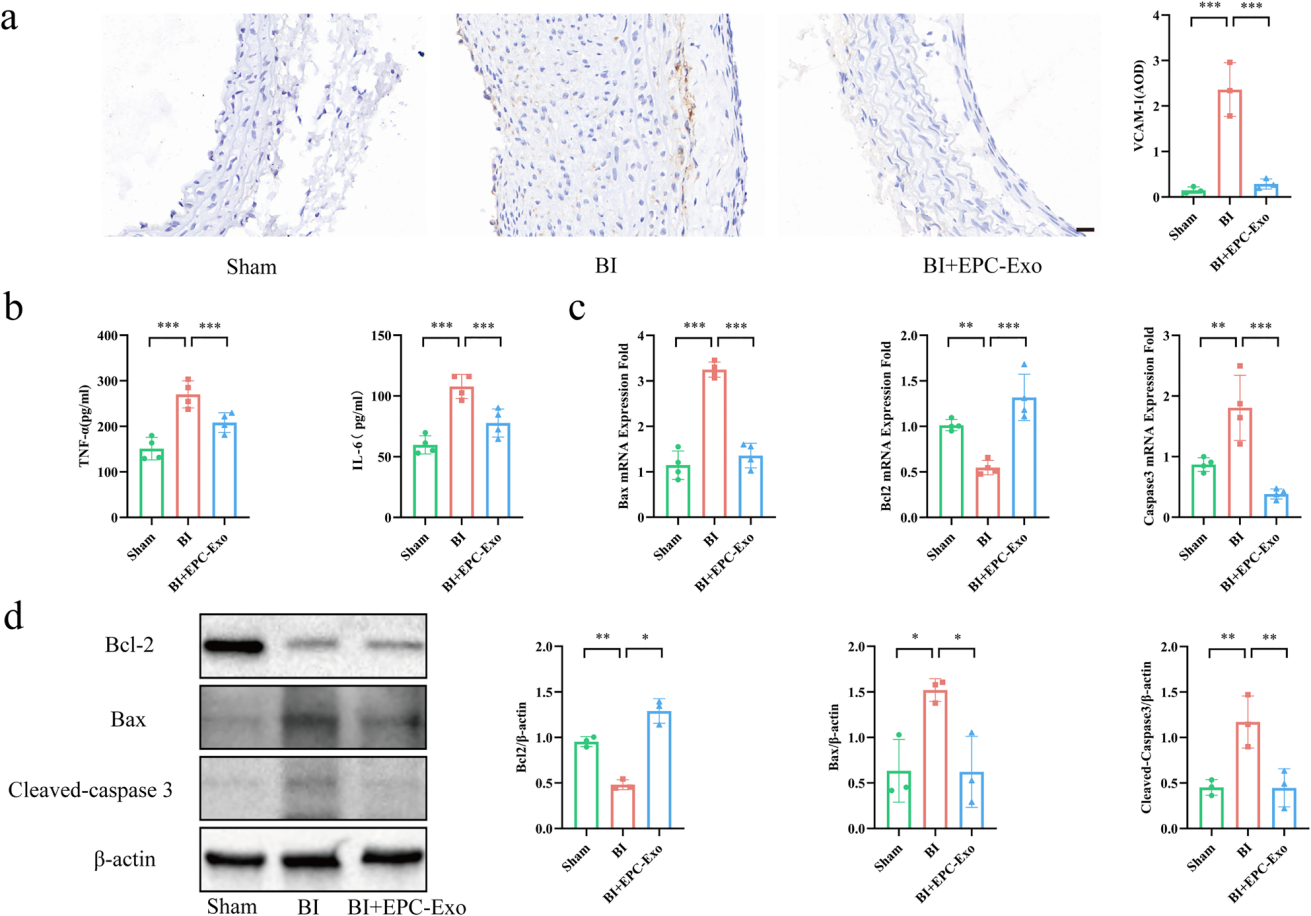
EPC-Exo alleviated inflammation and regulate the expression of genes and proteins relevant to apoptosis. (**a**) The expression of adhesion molecules on the vascular wall of carotid artery in rats were determined by immunohistochemistry. Representative images and quantitative data of VCAM-1 were shown. EPC-Exo could reduce adhesion molecule expression (scale bar = 20 μm). (**b**) Quantitative levels of inflammatory cytokines TNF-α and IL-6 in serum. EPC-Exo could inhibit the content of inflammatory factors in serum. (**c**) qPCR detected Bcl2, Bax, and Caspase 3 mRNA levels in the carotid artery. (**d**) Representative western blot images and quantitative data of Bcl2, Bax, and Cleaved-caspase 3 selection in carotid artery are shown. β-actin served as the reference protein (***p < 0.001, **p < 0.01, *p < 0.05, n ≥ 3 per group). The data are displayed as the M ± SD.

Vascular cell adhesion molecule-1 (VCAM-1), a cell surface protein typically expressed by endothelial cells, plays a crucial role in regulating the adhesion and migration of white blood cells, commonly employed as an indicator for assessing inflammatory status^19^. Immunohistochemistry revealed that both the vascular endothelium and middle membrane are visible to the (VCAM-1)-positive brown cell in BI model group, but the levels of these adhesion molecules were significantly decreased in the EPC-Exo group (Fig. 3a). The serum ELISA results revealed that the interleukin-6 (IL-6) and tumor necrosis factor-α (TNF-α) levels were significantly increased in serum after rat carotid artery BI (Fig. 3b). Notably, compared to the BI model group, VCAM-1 expression and concentration of inflammatory cytokine including IL-6 and TNF-α levels were significantly lower after EPC-Exo treatment. Associated genes and protein expression were also evaluated to explore the underlying mechanisms of the therapeutic effects of EPC-Exo treatment. According to qPCR analysis results, EPC-Exo treatment downregulated apoptosis-related genes, including the pro-apoptotic Bcl2-associated x (Bax) and Cleaved-caspase 3 genes (which were considered important cell apoptosis markers), and increased anti-apoptotic B-cell lymphoma-2 (Bcl2) gene expression (Fig. 3c). Consistent with qPCR detection, the WB assay revealed that EPC-Exo treatment affected the expression of apoptosis-related proteins (Fig. 3d- Supplementary material Figure S3).

These findings collectively show that EPC-Exo could decrease inflammation and regulate the expression of apoptosis-related genes.

### EPC-Exo enhanced endothelial function and stimulated VECs proliferation, migration, and tube formation in vitro

**Figure 4:**
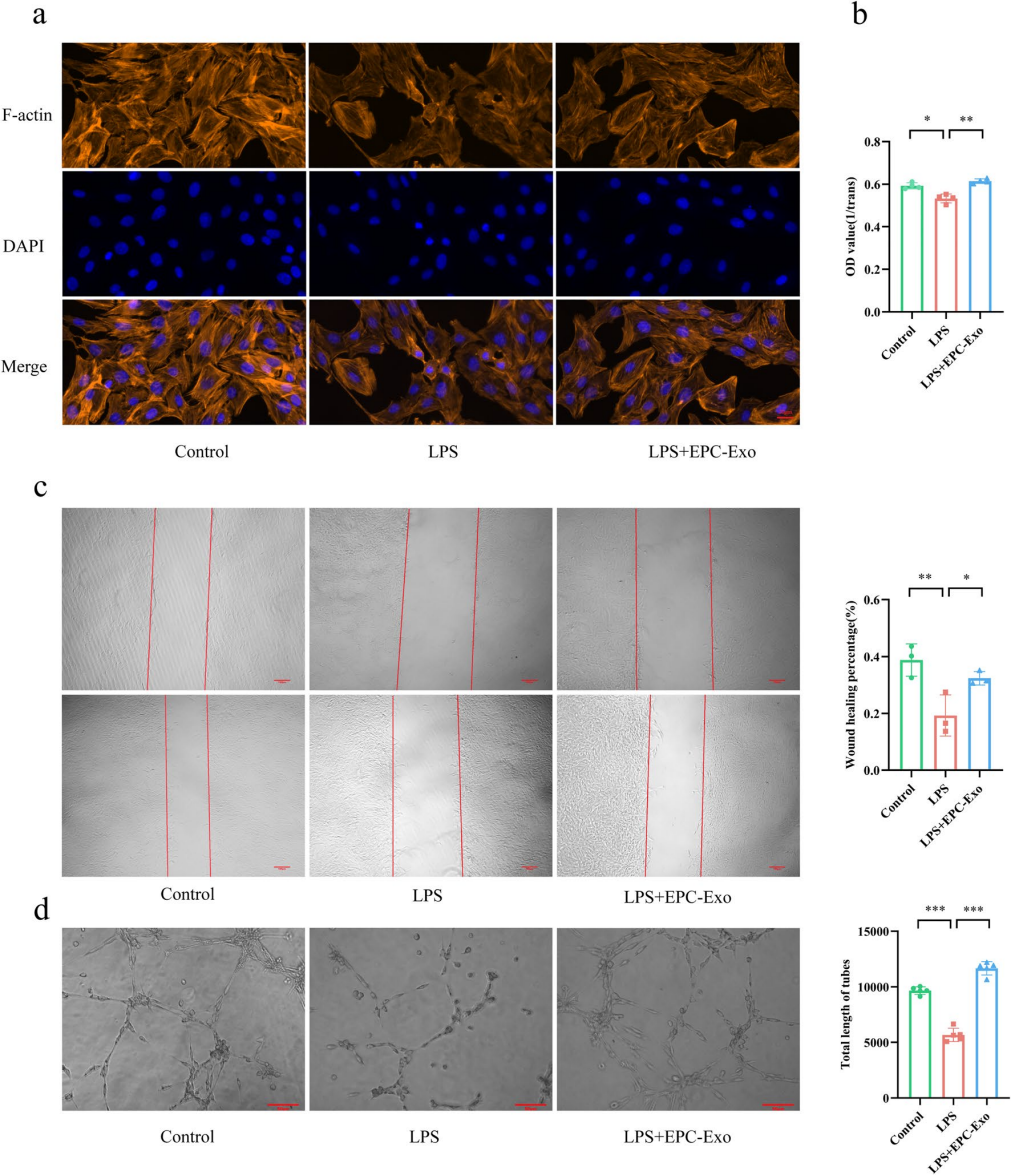
EPC-Exo enhanced endothelial function and stimulate the proliferation, migration and tube formation of VECs in vitro. (**a**) In actin cytoskeleton, the micro-silks constituted by fibrosactin (F-actin) were more complete, and the cell skeleton structure was obvious in EPC-Exo treatment group. (**b**) CCK-8 was used to detect the proliferation of VECs. The assay indicated that EPC-Exo stimulated the proliferation of VECs. (**c**) The scratch experiment revealed that EPC-Exo therapy could improve the capability of VECs to migrate after injury (scale bar = 100 μm). (**d**) In the tube formation assay, EPC-Exo increased the total length of tube of VECs (scale bar = 50 μm) (***p < 0.001, **p < 0.01,*p < 0.05, n ≥ 3 per group). The data are displayed as the M ± SD.

The in vitro ability of EPC-Exo to repair endothelial damage is still being investigated. Here, lipopolysaccharides (LPS) were incubated with VECs to establish endothelial damage models for evaluating VECs function, proliferation, migration, and tube formation after exosome treatment. The actin cytoskeleton forms the network of fibers within eukaryotic cells, serving as a crucial structural element. It is a valuable tool for observing cell morphology and assessing the extent of cellular damage. The actin cytoskeleton revealed a strong link between the cells as well as smooth and consistent micro-fibers constituting Fibrosactin (F-actin) in the control. However, the connections between cells were disrupted, and the micro-fibers were fractured in the LPS group. The actin cytoskeleton of VECs was largely restored after EPC-Exo treatment (Fig. 4a). The CCK-8 assay was used to determine the effect of EPC-Exo treatment on VECs proliferation, while the scratch and tube formation assays were performed to assess the VECs migratory and tune formation abilities. Compared to the control, LPS could suppress VECs proliferation, migration, and tube formation. However, these LPS-induced alterations were reversed by EPC-Exo therapy (Fig. 4b–d).

### EPC-Exo treatment reduced the LPS-induced VECs apoptosis and effectively regulated the activation of the Bcl2/Bax/Caspase-3 signaling pathway

**Figure 5:**
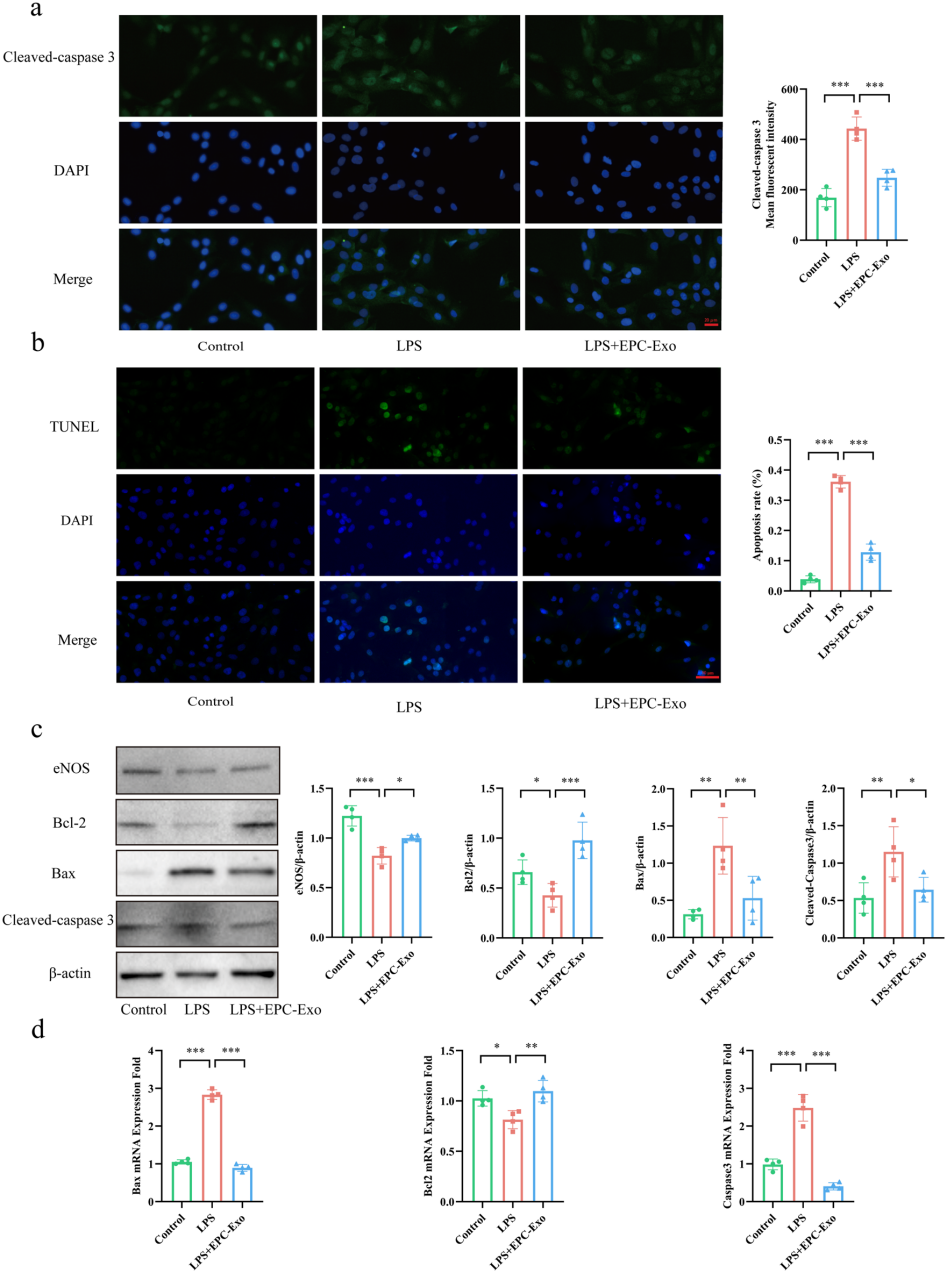
EPC-Exo decreased LPS-induced apoptosis of VECs and regulated the activation of the Bcl2/Bax/Caspase-3 signaling pathway. (**a**) Representative immunofluorescence images and quantitative data of Cleaved-caspase 3 expression in VECs were shown. EPC-Exo could decrease LPS-induced Cleaved-caspase 3 fluorescent intensity compared with the LPS group (scale bar = 20 μm). (**b**) By TUNEL staining, The VECs nucleus of LPS-induced combined green fluorescent dyes, which means cells were undergoing apoptosis. The amount of apoptosis VECs was decreased with EPC-Exo treatment (scale bar = 20 μm). (**c**) Representative western blot images and quantitative data of eNOS, Bcl2, Bax, Cleaved-caspase 3 selection in VECs were shown. (**d**) qPCR detected Bcl2, Bax, and Caspase 3 mRNA levels of VECs (***p < 0.001, **p < 0.01, *p < 0.05, n ≥ 3 per group). The data are displayed as the M ± SD.

An IF assay of Cleaved-caspase 3 along with transferase dUTP nick end labeling (TUNEL) staining was used to detect apoptosis in VECs. Through TUNEL staining, we observed that the LPS-induced VECs nuclei had mixed green fluorescent dyes, indicating that the cells were undergoing apoptosis. Subsequently, the IF assay demonstrated a substantial increase in the fluorescence intensity of cleaved-caspase 3 in the LPS group compared to the control. These findings showed that LPS could induce VECs apoptosis, whereas EPC-Exo treatment could lower the VECs apoptosis rate and Cleaved-caspase 3 protein expression (Fig. 5a,b). Additionally, WB and qPCR were used to detect the expression of related proteins and mRNA levels to explore the specific mechanism of EPC-Exo in inhibiting VECs apoptosis. The WB and qPCR analyses revealed that EPC-Exo treatment downregulated the apoptosis-related proteins and mRNAs, including Bax and Cleaved-caspase 3, but increased Bcl2 expression (Fig. 5c,d-Supplementary material Figure S5). These findings were consistent with those detected in vivo and collectively suggested that EPC-Exo can inhibit VECs apoptosis via the Bcl2/Bax/Caspase-3 signaling pathway.

## Discussion

Vascular endothelial damage is the primary etiological factor contributing to CVDs, thereby necessitating a strategic focus on endothelial injury repair and disease treatment, especially in advanced cases^20^. Although contemporary medical research presents promising preventive interventions like colchicine^21^, percutaneous coronary intervention (PCI) remains the principal technique for the treatment of coronary artery diseases (CADs)^2,22^. Despite the developments in CVDs treatment, persistent vascular restenosis after angioplasty remains a primary challenge in clinical practice due to its high recurrence rate. Therefore, it is imperative to explore novel therapeutic approaches to stabilize the PCI treatment efficacy for CVDs and effectively manage post-PCI complications. In this regard, clinicians can proactively monitor the sequelae of PCI surgery and achieve greater stability in CVDs management by identifying and implementing innovative treatment modalities.

Stem-cell therapy was previously believed to promote endothelial regeneration by replenishing and differentiating new cells^5^. However, subsequent research has revealed an alternative mechanism wherein EPCs primarily exert their vascular protective effects by secreting specialized factors, particularly exosomes^23^.

Exosomes are extracellular vesicles that have gained considerable attention in recent years and are commonly employed in CVDs treatment due to their potential anti-inflammatory, anti-apoptotic, and tissue regeneration-promoting actions^25–27^. Moreover, EPC-Exo have demonstrated remarkable efficacy in modulating inflammatory responses, enhancing survival rates, and minimizing acute lung damage. EPC-Exo can effectively enhance tissue perfusion when injected into the body and transplanted into the vasculature of ischemic tissues^28^. This remarkable therapeutic outcome underscores the potential of exosome-based treatment as a novel and promising cell-free vascular repair therapeutic option. According to recent pharmacological research, the primary mechanisms underlying the recurrence of vascular stenosis post-PCI involve the proliferation and migration of smooth muscle cells (SMCs) close to the endothelial injury site^29^. Hence, most medications employed to mitigate restenosis are aimed at inhibiting SMC proliferation. These findings provided the impetus for developing drug-eluting stents (DES)^30^. Interestingly, EPC-Exo primarily repair and restore endothelial cells by promoting their proliferation and migration, and facilitating repair of vascular endothelium^31^. These findings have been established in various studies and reinforce the potential significance of EPC-Exo in vascular regeneration and therapeutic interventions.

In this study, EPCs were extracted from rats, and their purity was meticulously determined. After acquiring high-purity EPCs, the exosomes released by these cells were extracted and identified. A rat carotid artery BI model was constructed by inducing endothelial damage in rats after feeding them with high-fat diet for two weeks. The BI model group exhibited noticeable signs of vascular stenosis, disrupted endothelial cell structure, and vascular neointimal hyperplasia. Furthermore, EPC-Exo demonstrated remarkable therapeutic potential in this model. Specifically, these exosomes adhered to the damaged vascular endothelium, effectively enhancing endothelial function and inhibiting excessive endothelial hyperplasia thereby impeding vascular restenosis.

**Figure 6:**
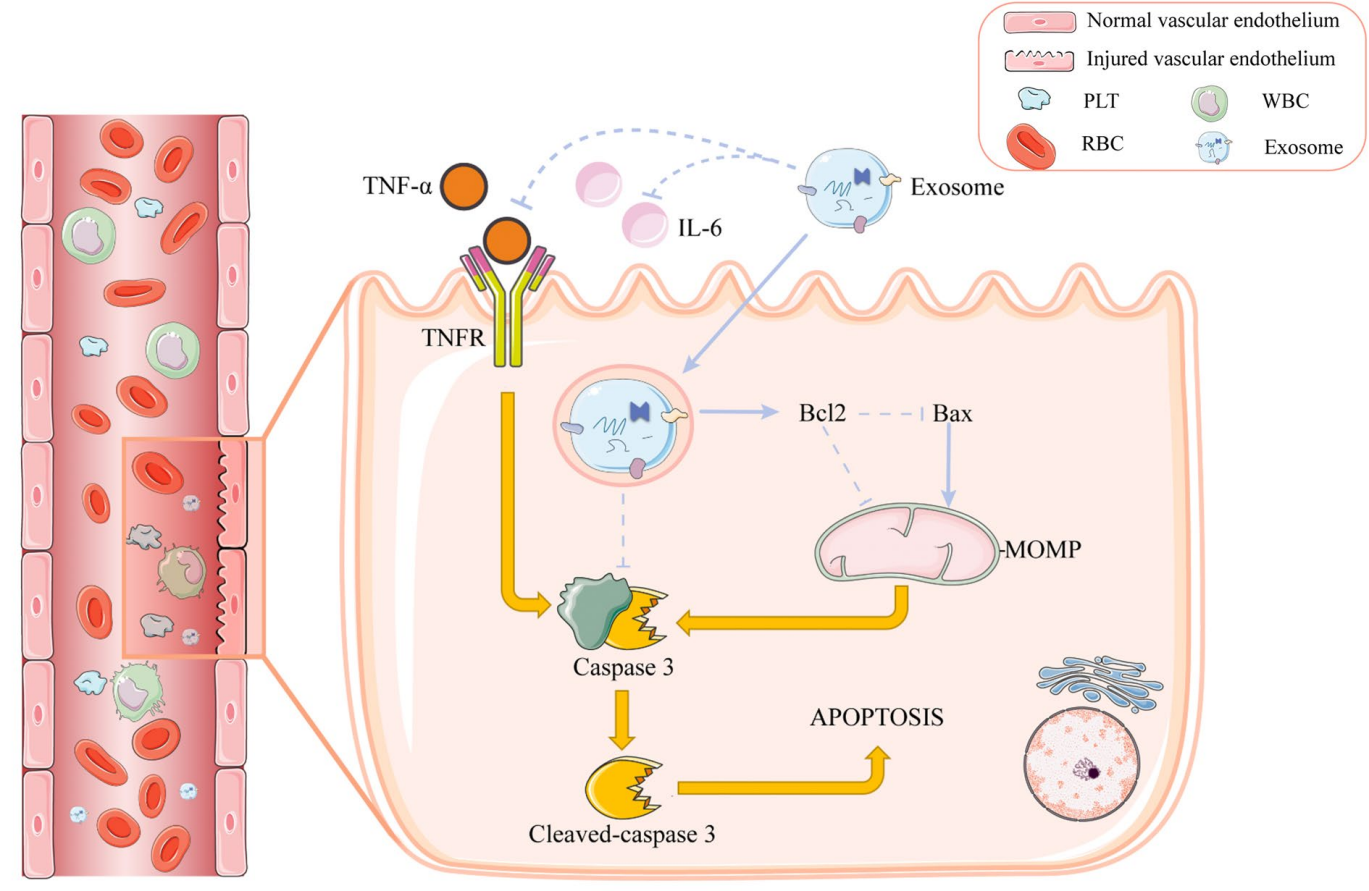
Hypothesis figure. Effects of EPC-Exo on Bcl2/Bax/Caspase-3 signaling pathway in rat vascular endothelial cells.

Numerous factors influence vascular reendothelialization post-injury, among which post-PCI inflammation and endothelial cell apoptosis induced by various factors are the most critical. However, the apoptosis of injured cells and the underlying mechanisms involved in this process have not been comprehensively explored. Besides PCI-induced acute inflammation, direct endothelial cell injury and the release of inflammatory elements in the plaque also frequently trigger inflammatory responses^32^. Apoptosis is primarily defined as programmed cell death^33^, which is mainly characterized by chromatin condensation, cell constriction, and the formation of apoptotic bodies, and is regulated by various chemicals and proteins^34,35^. There are two main apoptotic pathways: intrinsic and extrinsic^36^. The Bcl2 family of proteins controls mitochondrial permeability to various proteins as well as mitochondrial outer membrane permeabilization (MOMP)^37^, playing a crucial role in regulating the intrinsic mitochondrial apoptotic pathway^38^. Although Bcl2 was initially identified as a cancer gene, its anti-apoptotic properties were found to enhance B-cell lymphoma proliferation^39^. However, Bcl2 protein does not impair normal cell proliferation control, but enhances cell survival by inhibiting programmed cell death. Meanwhile, Bax, the pro-apoptotic member of the Bcl2 family, increases mitochondrial membrane permeability, thereby releasing apoptotic factors into the cytoplasm and ultimately activating Caspase-3, which, in turn, leads to apoptosis^36^. These two protein-mediated apoptosis pathways constitute the intrinsic mitochondria-mediated apoptosis pathway. The extrinsic apoptosis pathway is triggered after cell damage when death signals are received following stimulation by exogenous inflammatory cytokines, such as TNF-α. These cytokines interact with membrane receptors TNFR and TLR4, triggering a series of reactions that eventually lead to cell apoptosis^36^. Overall, we deduced that Caspase-3 is the executor of cell apoptosis, and that it is activated by the above-mentioned intrinsic and extrinsic apoptosis pathways to initiate cleavage, ultimately mediating apoptosis (Fig. 6).

In this study, the levels of adhesion molecules and inflammatory factors were also measured. Their concentrations increased after carotid artery BI, but this effect was reversed by EPC-Exo treatment. These findings suggest that EPC-Exo can mitigate the initiation of the inflammatory factor-mediated extrinsic apoptosis pathway. Furthermore, EPC-Exo were found to suppress the expression of genes and proteins associated with the intrinsic apoptosis pathway. In vitro experiments were conducted to examine the mechanisms underlying EPC-Exo therapy.

We treated VECs with LPS to establish an in vitro endothelial cell oxidative damage model. The LPS-induced damage disrupted cell connections and caused microfilament fractures. However, EPC-Exo treatment mitigated the LPS-induced damage, preserving actin cytoskeleton integrity. Additionally, EPC-Exo treatment enhanced VECs proliferation, migration, and tube formation functions. Furthermore, LPS increased the VECs apoptosis rate and upregulated the apoptosis-related proteins. However, EPC-Exo treatment reversed these effects, lowering the apoptosis rate by down-regulating the apoptosis-related proteins, Bax, and Caspase-3 but up-regulating Bcl2. According to the Bcl2/Bax/Caspase-3 protein and gene expression analysis results, EPC-Exo treatment could suppress the signaling pathways involved in the intrinsic apoptosis cascade. The results of the in vitro and in vivo experiments suggest that the EPC-Exo-induced endothelial apoptosis inhibition is mediated through the intrinsic and extrinsic apoptosis pathways, which involve the Bcl2/Bax/Caspase-3 signaling pathway. In summary, combining in vitro and in vivo strategies, EPC-Exo could inhibit excessive carotid intimal hyperplasia after BI, promote the repair of endothelial cells in intimal injury, and enhance endothelial function. The underlying mechanism involves the suppression of inflammation and anti-apoptotic effects. The fundamental mechanism for this anti-apoptotic effect involves the regulation of the Bcl2/Bax/Caspase-3 signaling pathway. This study illuminates the mechanism for preventing vascular restenosis through apoptosis regulation, providing a novel perspective on EPC-Exo treatment in repairing vascular endothelial injuries by targeting VECs. Moreover, this study elucidates the mechanisms and therapeutic effects of exosomes, which not only enhance our understanding of their multifaceted roles in vascular biology but also offer an avenue for developing innovative and effective cell-free therapeutic approaches for vascular regeneration. However, this study had some limitations. The exact components of exosomes responsible for therapeutic effects, such as proteins, lipids, and nucleic acids, remain unknown. Furthermore, this study did not explore the specific mechanisms underlying the inhibition of the inflammatory response by exosomes. Moving forward, it is imperative to address these gaps by elucidating the specific substances through which exosomes exert therapeutic effects and clarify the mechanisms involved in the suppression of the inflammatory response.

## Materials and methods

### Experimental materials

Rat bone marrow endothelial progenitor cells separation kits were bought from TBD company (China). CD34, and CD133 were purchased from Abcam (UK). Bax and Bcl2 were purchased from Proteintech (China). VCAM-1 was bought from Abcam (UK). ELISA kits for ET-1, TNF-α, IL-6 were purchased from ZCibio (China). Transferase dUTP nick end labeling (TUNEL) staining kit was purchased from Beyotime (China). Cleaved-caspase3 was purchased from Affinity (China). Rat bone marrow-derived endothelial progenitor cells complete medium was purchased from Procell (China). Endothelial cell growth medium-2 (EGM-2) was purchased from Lonza (Switzerland). LPS was purchased from Sigma (US). Mouse Monoclonal CD34 Antibody and Rabbit Polyclonal CD133 Antibody, used to flow cytometry, were bought from Univ (China). Cell culture medium was purchased from Pricella (China). Human Dil-labeled acetylated low-density lipoprotein (Dil-LDL) was purchased from MKbio (China). FITC labeled ulex europaeus agglutinin 1 (FITC-UEA-1) was purchased from Yiyuan Biotechnology (China). Ultrafiltration centrifugal tubes were purchased from Millipore (Germany). Ultracentrifuge tubes were purchased from Beckman (US).

### Methods

#### Animal

All animal experiments were ethically approved by the Animal Experiment Center of Hunan University of Chinese Medicine (HNUCM) Ethics Committee (Approval number: LL2022091403). All experiments were performed in compliance with national and institutional laws. The acquisition and description of data followed the recommendations are reported in accordance with the ARRIVE guidelines. All animals were kept in the barrier system at the animal experiment center of HNUCM at a controlled temperature and humidity. The rats were randomly categorized into different treatment groups. All surgeries and follow-up analyses were performed through a blinded intervention approach.

#### Cell isolation, culture, and exosome extraction

First, Sprague–Dawley (SD) rats weighing 100–120 g were euthanized by CO2 inhalation and immersed in 75% alcohol for 15 min to achieve disinfection in preparation for EPCs extraction. Following the manufacturer's instructions, the EPCs isolation kit was used to extract EPCs, which were then cultured in rat bone marrow-derived endothelial progenitor cells complete medium in a 37 °C and 5% CO2 incubator. After cell passaging, the medium was changed to endothelial cell growth medium-2 (EGM-2). For exosome extraction, 70–80% confluent EPCs were washed with phosphate-buffered saline (PBS) and changed to a fresh exosome-depleted culture medium. After culturing for 24 h, the medium was collected in centrifuge tubes. The medium containing exosomes was extracted through high-speed centrifugation combined with ultrafiltration. The supernatant was subjected to gradient centrifugation at 4 °C: 500×*g* for 25 min, 3000×*g* for 15 min, and 12,000×*g* for 30 min, aiming to remove cellular debris, apoptotic vesicles, and macrovesicles. The cell-free supernatant, cleared of impurities, was then transferred into a 30kD ultrafiltration tube and centrifuged at 3000×*g* for 25 min to collect exosomes. The resulting precipitate was further purified through centrifugation for 90 min at 120,000×*g*. Thereafter, the precipitate was resuspended in 200 μL PBS and stored at − 80 °C for further analysis.

VECs were extracted and identified following the methods outlined in previous research^40^, then cultured in Dulbecco’s modified eagle's medium/nutrient mixture F-12 (DMEM/F12) medium [with 10% fetal bovine serum (FBS), 100 U/ml penicillin, and 100 μg/ml streptomycin] in a 37 °C and 5% CO2 incubator.

#### Establishment of carotid BI model

15 Male SD rats [Special Pathogen-Free (SPF) weighing 300–350 g; aged 6–8 weeks] were purchased from Hunan Slaughter Jingda Laboratory Co.S, Ltd. (Animal license number: SCXK (Xiang) 2019–0004). High-fat chow was purchased from Beijing Keo Collaborative Feed, and the formulation was prepared as reported in our previous study^41^. The animals were fed with high-fat chow for two weeks. The experimental method of establishing carotid BI model was described previously^42^. The rats were anesthetized with 2.5% pentobarbital and injected with penicillin three days post-surgery to prevent infection. The rats in the sham group had only their carotid arteries isolated. All rats with established carotid BI were simultaneously randomized into the BI model group and the EPC-Exo group. The exosome group animals were injected with EPC-Exo (30 μg) 12 h post-surgery and on the third day after the operation, while the other groups were injected with an equal volume of PBS. The rats were euthanized by CO_2_ inhalation after 14 days. Half of the carotid arteries were fixed in 4% paraformaldehyde and the other half were stored at − 80 °C.

#### Delivery of exosomes

**Figure 7:**
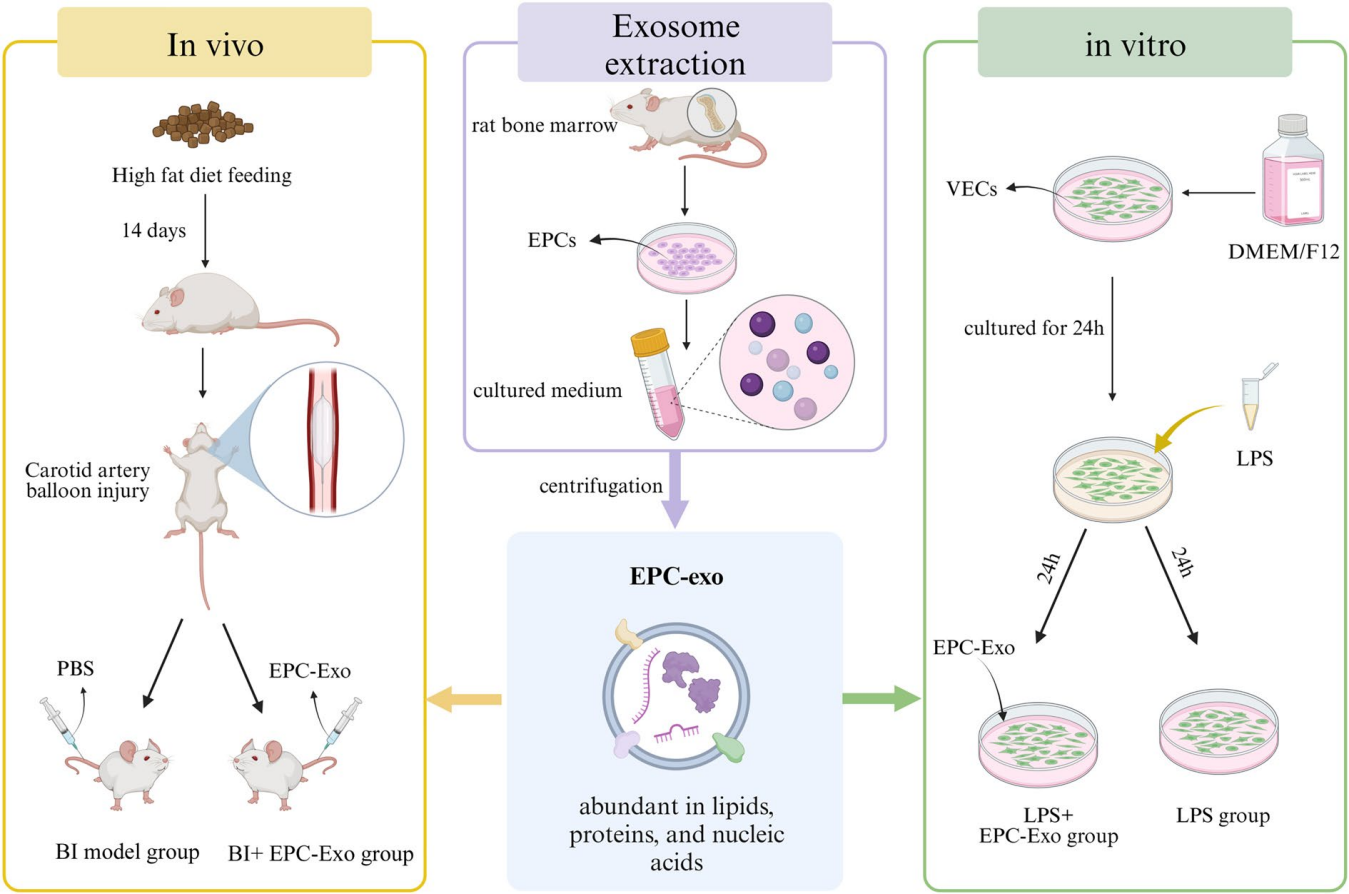
Animal and cell models establishment and processing flowchart. Figure created with BioRender (https://biorender.com/).

To monitor the internalization of EPC-Exo, the purified exosomes were labeled using an exosomal red fluorescent labelling dye (PKH26) kit. The exosome group animals were injected with PKH26-labelled EPC-Exo ( 30 μg) 12 h post-surgery and on the third day after the operation, while the other groups were injected with an equal volume amount of PBS (Fig. 7). Whether EPC-Exo adhered to the injured carotid artery vessel wall after injection and the binding of exosomes were assessed by fluorescence. After seven days of modeling, the rats in the model and EPC-Exo groups were euthanized by CO_2_ inhalation. The intact carotid arteries were extracted and fixed in paraformaldehyde. Subsequently, the fixed carotid arteries were dehydrated, made transparent, embedded and made into paraffin sections. Nuclei were counterstained with 4′,6-diamidino-2-phenylindole (DAPI). The sections were visualized and imaged under a fluorescence microscope, focusing on the intimal area of the carotid artery. Image J software was used to analyze the fluorescence intensity.

#### Flow cytometry

The EPCs were centrifuged two times at 1000 rpm to remove the residual medium and then resuspended in PBS. Subsequently, the cells were incubated with CD34 (1:100) and CD133 (1:100) antibodies and left on ice for 30 min. Following incubation, the cells were centrifuged and resuspended in 200 μl PBS, and the results were analyzed by DxP AthenaTM flow cytometry (Cytek, US).

#### Transmission electron microscope

The exosome solution was first added dropwise to the copper grid and incubated for 5 min at room temperature (RT) before blotting the excess solution with filter paper. Subsequently, 10 μL of 2.5% glutaraldehyde solution was added dropwise to the copper grid for 10 min and washed with PBS 1–2 times for 3 min each. Following that, the surface was washed once with PBS and allowed to dry at RT. Finally, transmission electron microscopy was used to examine the morphology of the exosomes.

#### Exosome particle size analysis

To avoid clogging the injection needle, the exosomes were removed and diluted to the appropriate multiple with a PBS gradient. After verifying the accuracy of the particle size and concentration analyzer against set standards, we tested the sample by placing it on the machine and obtained information on exosome particle size and concentration.

#### Hematoxylin and eosin (H&E) staining

To assess intimal hyperplasia after endovascular injury, the sections were stained with H&E staining, focusing on the endovascular area. The fixed vessels were dehydrated, made transparent, embedded, sectioned, and then stained using hematoxylin and eosin. They were then photographed under the microscope to be sealed and dried. Three fields of view (40×) were randomly selected under the microscope, and the mean value was taken as the measurement. The intramembranous area of the inner outer elastic membranes, lumen and their perimeters were analyzed using Image Pro Plus software 6.0. The Intima-media thickness (IT) and hyperplasia ratio of intima thickness (HRIT) were calculated based on previous studies^41^. The calculations are as follows: IT = (perimeter of the internal elastic tunica − perimeter of the lumen)/2π; MT = (perimeter of the external elastic tunica − perimeter of the internal elastic tunica)/2π; HRIT = IT/(IT + MT) × 100%.

#### Immunofluorescence (IF)

To identify the EPCs, cultured EPCs were fixed in 4% paraformaldehyde for 30 min. After washing with PBS, the cells were blocked with 3% bovine serum albumin (BSA) for 60 min. The EPCs were then incubated with primary antibodies CD34 (1:500) and CD133 (1:500) at 4 °C overnight. After incubation, the cells were washed with PBS and then stained for 60 min with a fluorescent secondary antibody (1:500) at RT in the dark. Thereafter, the nuclei were stained with DAPI, and the EPCs were examined by immunofluorescence (IF). EPCs have the ability to take up Dil-LDL and bind fluorescein isothiocyanate (FITC)-UEA-1. Cultured EPCs were added to complete medium containing Dil-LDL and cultured in a 37 °C and 5% CO_2_ incubator for 4 h. The cells were then fixed in 4% paraformaldehyde for 20 min before adding PBS containing FITC-UEA-1 and allowing the mixture to settle at RT for 2 h. Subsequently, the EPCs were stained with DAPI for 6 min. The cells were then fixed in 4% paraformaldehyde for 20 min before adding PBS containing FITC-UEA-1 and allowing the mixture to settle at RT for 2 h. Subsequently, the EPCs were stained with DAPI for 6 min.

To investigate the expression of apoptosis-related proteins in different groups of VECs, the VECs were fixed with 4% paraformaldehyde for 30 min for IF testing. Cultured VECs were blocked with 3% bovine serum albumin (BSA) for 60 min, and then incubated with Cleaved-caspase-3 primary antibody at 4 °C overnight. Thereafter, the VECs were washed and incubated with FITC-conjugated secondary antibody for 1 h. Nuclei were stained with DAPI. All cells were sealed with an anti-fluorescence quenching sealer and photographed under a fluorescence microscope. Image J software was used to analyze the fluorescence intensity.

#### Immunohistochemistry

Paraffin sections were first dewaxed with water and then antigenically repaired through dropwise addition of an Antigen Repair Solution. Subsequently, the sections were incubated in a 3% hydrogen peroxide solution for 25 min at RT to block the endogenous peroxidase. The sections were then incubated overnight at 4 °C in a wet box with a drop of 3% BSA at RT for 30 min. Primary antibodies [VCAM-1 (1:100)] were then added to the sections. After incubation, the cells were washed before adding a freshly prepared diaminobenzidine (DAB) coloring solution dropwise onto the sections under the microscope for a controlled period. The hematoxylin-stained nuclei were sealed with neutral resin. Three fields of view (40×) were randomly selected under the microscope, and the mean value was taken as the measurement. Pictures were captured under a microscope after drying the sections. Image J software was used to analyze the positive area: Mean density = sum of integral optical density (IOD)/area of measurement area.

#### Enzyme‑linked immunosorbent assay (EILISA)

The rat serum was obtained and left to stand at 4 °C for two hours, then centrifuged at 3000 rpm for 15 min. The serum was divided and stored at − 80 °C. According to the instructions of the ELISA kit, the levels of ET-1, IL-6, and TNF-α in rat serum was determined.

#### Cell treatment and proliferation

The cell counting kit-8 (CCK-8) assay was used to detect VECs viability. The VECs were seeded in 96-well plates at a density of 1 × 10^5^ cells/ml. The VECs in the control group were treated with a complete ordinary medium for 24 h, whereas cells in other groups were treated with LPS (1 mg/ml) for 24 h, as outlined in previous research^43^, to establish an endothelial cell injury model. Furthermore, the control and LPS groups were treated by replacing their culture media with fresh culture media, while the exosome group was treated with a complete culture medium containing exosomes for 24 h (Fig. 7). According to the treatment of cells, the cells were divided into three groups: control (added to complete medium), LPS [both added to complete medium containing LPS (1 μg/ml)], and LPS + EPC-Exo [added to a complete medium containing LPS (1 μg/ml) and complete medium containing EPC-Exo (10 μg/ml)]. Subsequently, a 10% CCK-8 solution was added, and the mixture was placed in the incubator before measuring the absorbance values at a wavelength of 420 nm with an enzyme marker for 1–2 h.

#### Scratch experiment

VECs were inoculated into 6-well plates (2 × 10^5^ cells/well). After the cells had adhered, a straight line was drawn vertically and evenly in the middle of the well plate with the tip of a gun. The cells were washed with PBS to remove the scratched cells, the control group was treated with regular medium, while the other groups were treated with normal medium containing LPS (1 μg/ml) for 24 h. The cells were incubated at 37 °C in a 5% CO2 incubator. The same location was photographed at 0 h and 24 h. The scratch experiment were observed using an Aiovert A1 inverted microscope (Zeiss, Germany). Respectively, the area before and after injury was analyzed by ImageJ.

#### Tube formation assay

The matrigel solution was allowed to melt overnight at 4 °C, and the 96-well plates and 200 μl tips were pre-cooled at − 20 °C. The thawed matrigel solution was then added to the 96-well plates on the following day and placed in the incubator for 30 min. Subsequently, 100 μl of cells (1 × 10^5^ cells/ml) were added to the wells. The cell tube formations were observed using an Aiovert A1 inverted microscope (Zeiss, Germany), and three randomly selected fields of view were photographed and counted.

#### Transferase dUTP nick end labeling (TUNEL) staining

The cells were treated as previously described. Following VECs fixation, PBS containing 0.3% Triton X-100 was added, and the mixture was incubated for 5 min at RT. The cells were then washed two times with PBS after incubation. The TUNEL assay was performed using a TUNEL assay kit per the manufacturer's instructions. After preparation, 50 μl of the TUNEL assay solution was added to the samples and incubated for 60 min at 37 °C. During cultivation, an appropriate amount of water should be added to the excess well space to keep it moist, thereby minimizing the TUNEL determination solution's evaporation. After washing, the nuclei were stained with DAPI for 6 min before sealing with an anti-fluorescence quenching blocking solution and observing under a fluorescence microscope.

#### Actin cytoskeleton staining

Actin cytoskeleton staining using phalloidin is commonly used to study the morphology and integrity of the network of fibers of the cells, which causes the skeleton fiber filaments to exhibit red fluorescence. Cell treatment was performed as previously described. The VECs were fixed and blocked with a 3% BSA solution for 30 min. After washing with PBS three times, 250 μl Phalloidin (1:500) was added and incubated for 1 h at RT. The cells were then stained with DAPI for 6 min. Subsequently, the cells were sealed with an anti-fluorescence quencher after washing three times with PBS and observed under a fluorescent microscope.

#### Fluorescent quantitative PCR (qPCR)

Following the standard protocols, the total RNA kit was used to extract total RNA from carotid tissues and VECs. The RNA was reverse transcribed to synthesize cDNA and then amplified by PCR. Glyceraldehyde-3-phosphate dehydrogenase (GAPDH) was used to evaluate and standardize the relative gene expressions through the 2 − ^ΔΔ^Ct method. Table 1 shows the primer sequences used.

**Table 1:** The primer sequences of target genes.

Target gene	Primer sequences
Bcl2	Forward:5′-TGACTTCTCTCGTCGCTACCGT-3′
	Reverse: 5′-CCTGAAGAGTTCCTCCACCACC-3′
Bax	Forward: 5′-GCCTTTTTGCTACAGGGTTTCAT-3′
	Reverse: 5′-TATTGCTGTCCAGTTCATCTCCA-3′
Caspase-3	Forward: 5′-TGGAATGTCATCTCGCTCTGGT-3′
	Reverse: 5′-GAAGAGTTTCGGCTTTCCAGTC-3′
GADPH	Forward: 5′-ACGGCAAGTTCAACGGCACAG-3′
	Reverse: 5′-CGACATACTCAGCACCAGCATCAC-3′

#### Western blot (WB) analysis

Rat carotid arteries were transferred into a grinding tube and finely ground before lysing on ice for 30 min to extract the supernatant. Subsequently, Sodium Dodecyl Sulphate (SDS) was added to determine and denature the proteins. Cell and exosome proteins were extracted as previously described without grinding. The proteins were then separated through polyacrylamide gel electrophoresis, transferred to a PVDF membrane, blocked with 10% milk for 1 h, and incubated overnight at 4 °C with primary antibodies CD63 (1:2000), tumor susceptibility gene 101 protein (TSG101) (1:1000), CD81 (1:1 000), Calnexin (1:1000), eNOS (1:500), Bcl2 (1:2000), Bax (1:2000), β actin (1:8000). After washing 3 times with TBS-Tween (TBST), secondary antibodies (1:10,000) were incubated at room temperature for 1 h. Bands were detected by GelDocXR + Gel Imaging System (BioRad, USA) and analyzed by Image J software.

#### Statistical analysis

All statistical analyses were performed using SPSS software. The data were quantitative and continuous, and the statistics for each test were expressed as mean ± standard deviation (M ± SD). One-way analysis of variance (ANOVA) and the least significant difference (LSD) test were used to compare groups if the normality and homogeneity of variance were met. On the other hand, the Dunnet T3 test was employed when variance was not homogeneous.

### Supplementary Information

Supplementary Figures.

## Data Availability

The datasets used and/or analyzed during the current study are available from the corresponding author upon reasonable request.

## Abbreviations

CVDs: Cardiovascular disease
BI: Balloon injury
EPC: Endothelial progenitor cells
EPC-Exo: Exosomes derived from endothelial progenitor
VECs: Vascular endothelial cells
SD: Sprague–Dawley
Exo: Exosomes
Exo-PKH26: PKH26-labelled EPC-Exo
Dil-LDL: Human Dil-labeled acetylated low density lipoprotein
FITC-UEA-1: FITC labeled ulex europaeus agglutinin I
TSG101: Tumor susceptibility gene 101 protein
TNF-α: Tumor necrosis factor-α
IL-6: Interleukin-6
VCAM-1: Vascular cell adhesion molecule-1
ET-1: Endothelin-1
eNOS: Endothelial nitric oxide synthases
CAD: Coronary artery diseases
IT: Intimal thickness
HRIT: Hyperplasia ratio of intima thickness
PCI: Percutaneous coronary intervention
SDS: Sodium dodecyl sulphate
F-actin: Fibrosactin
GADPH: Glyceraldehyde-3-phosphate dehydrogenase
Bcl2: B-cell lymphoma-2
Bax: Bcl2-associated x
SMC: Smooth muscle cells
MOMP: Mitochondrial outer membrane permeabilization
LPS: Lipopolysaccharides
WBC: White blood cells
RT: Room temperature
DES: Drug-eluting stents
DMEM/F12: Dulbecco's modified eagle's medium/nutrient mixture F-12
FBS: Fetal bovine serum
SPF: Special Pathogen-Free
PBS: Phosphate buffer saline
CCK-8: The cell counting kit-8
EILISA: Enzyme-linked immunosorbent assay
HE: Hematoxylin and eosin
IF: Immunofluorescence
TUNEL: Transferase dUTP nick end labeling
DAPI: 4′,6-Diamidino-2-phenylindole
BSA: Bovine serum albumin
WB: Western blot
LSD: Least significant difference
ANOVA: Analysis of variance
