# Supplementary material for: Exosomes of endothelial progenitor cells repair injured vascular endothelial cells through the Bcl2/Bax/Caspase-3 pathway
Source: Sci Rep. 2024 Feb 23;14:4465. doi: 10.1038/s41598-024-55100-x (PMC10891177; doi:10.1038/s41598-024-55100-x)

**Supplementary data Original blots**

**Figure 1f**


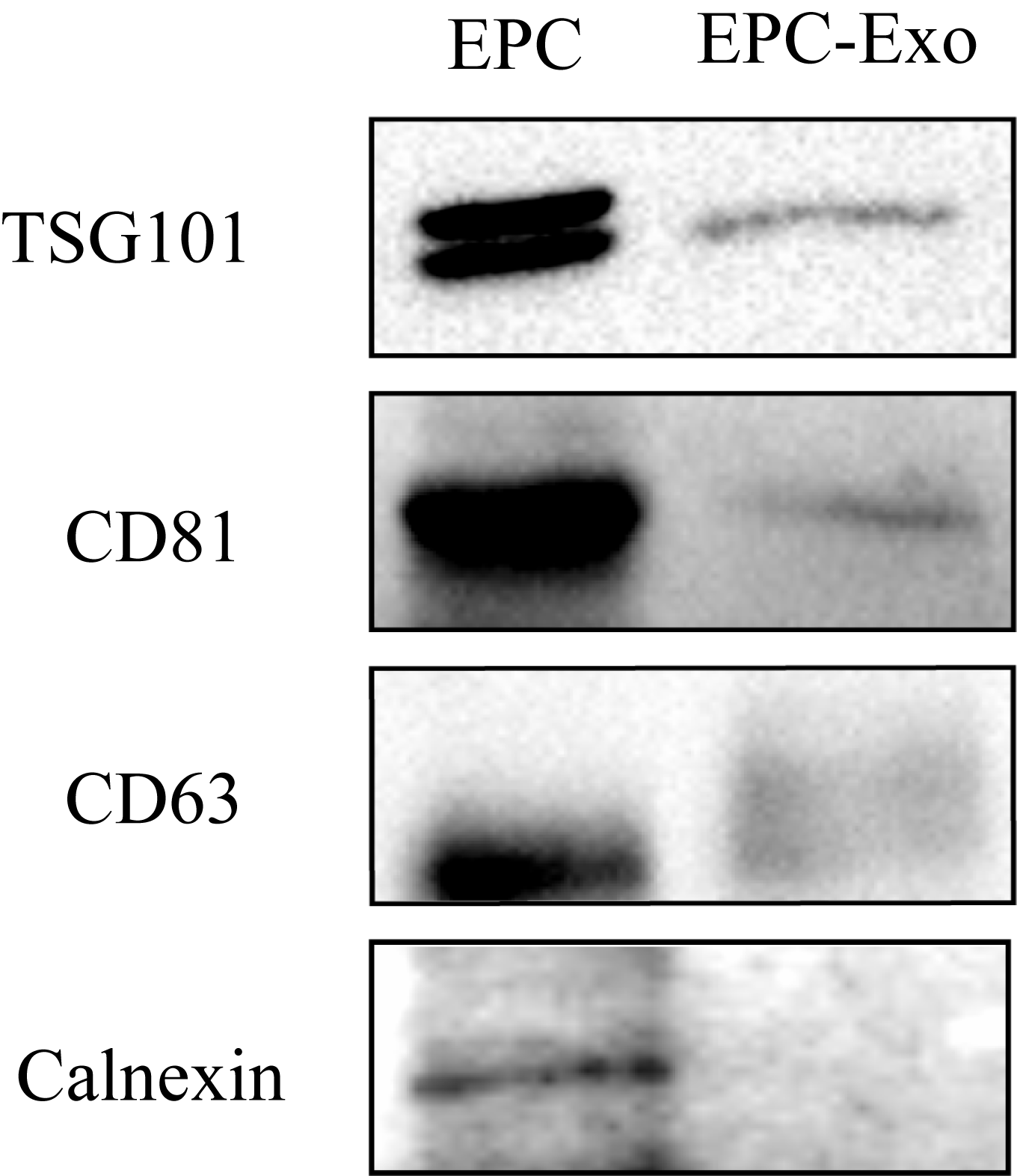


1

2

3

4

For Figure 1f, a replicate of the

experimental data shown in the

manuscript is provided.

1 2


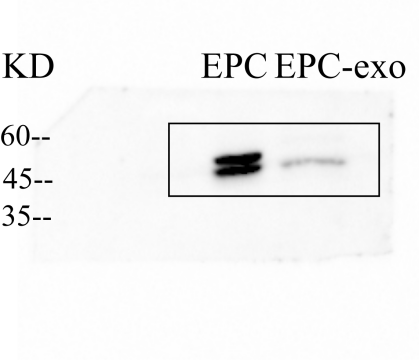

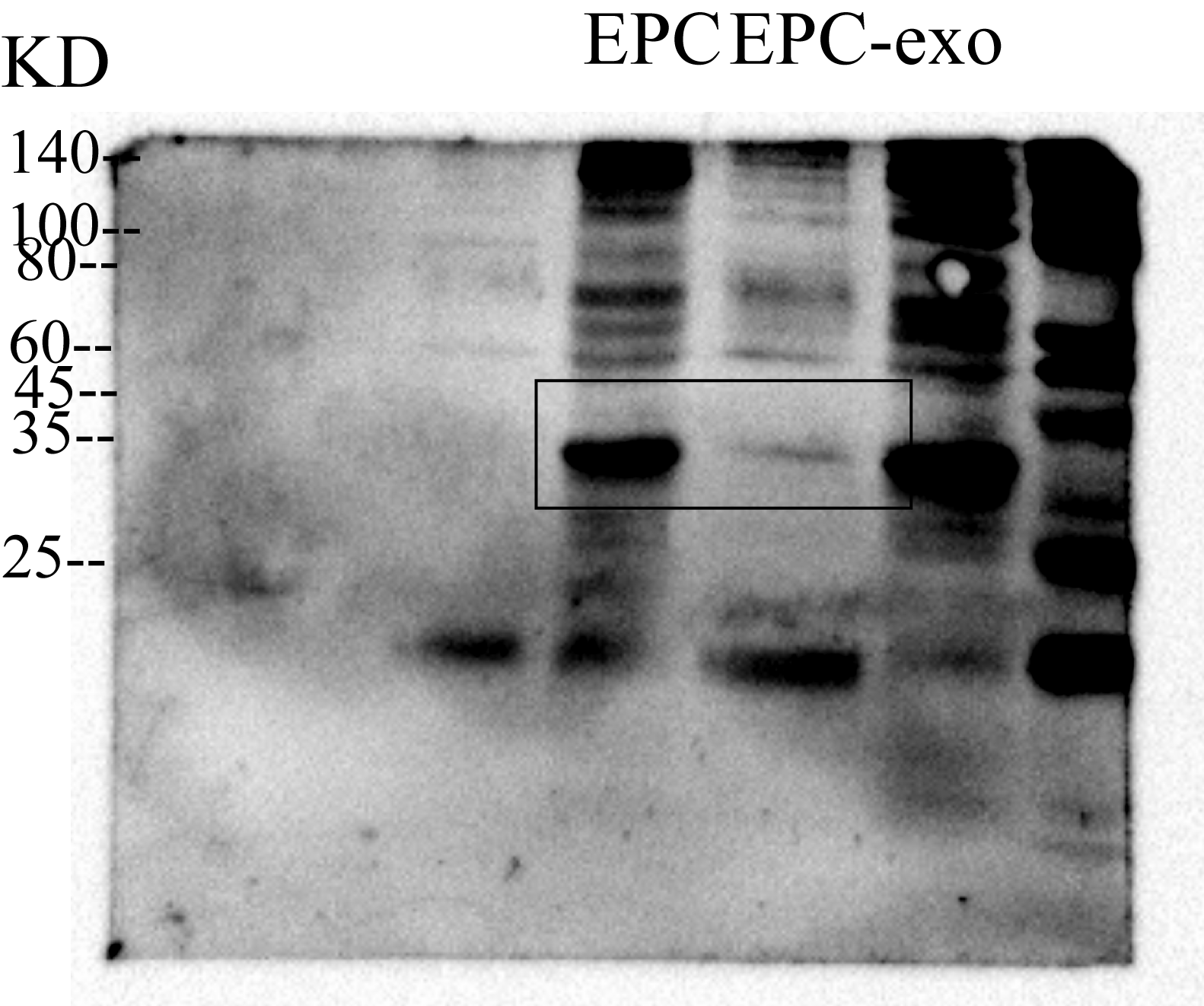


3 4


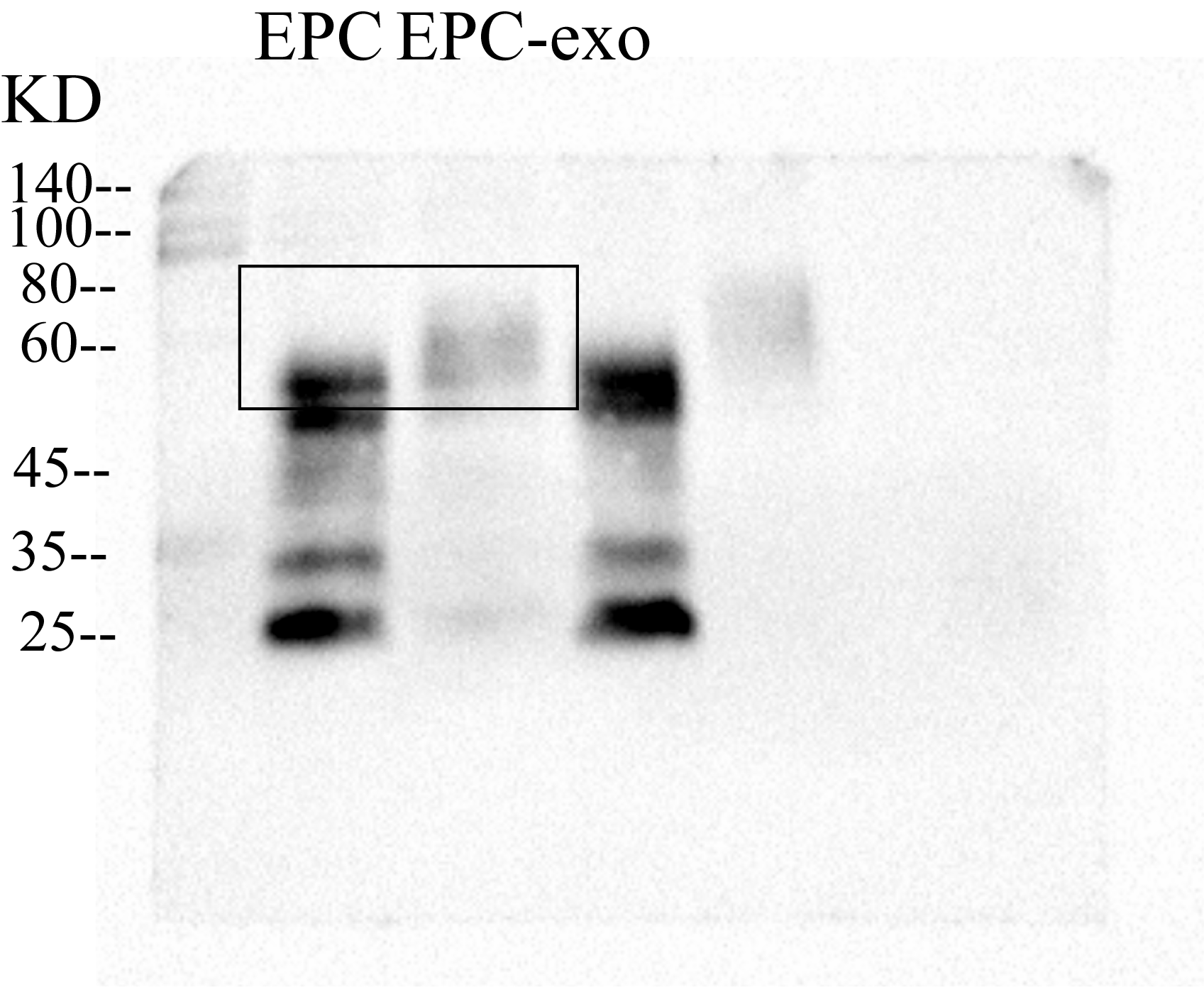

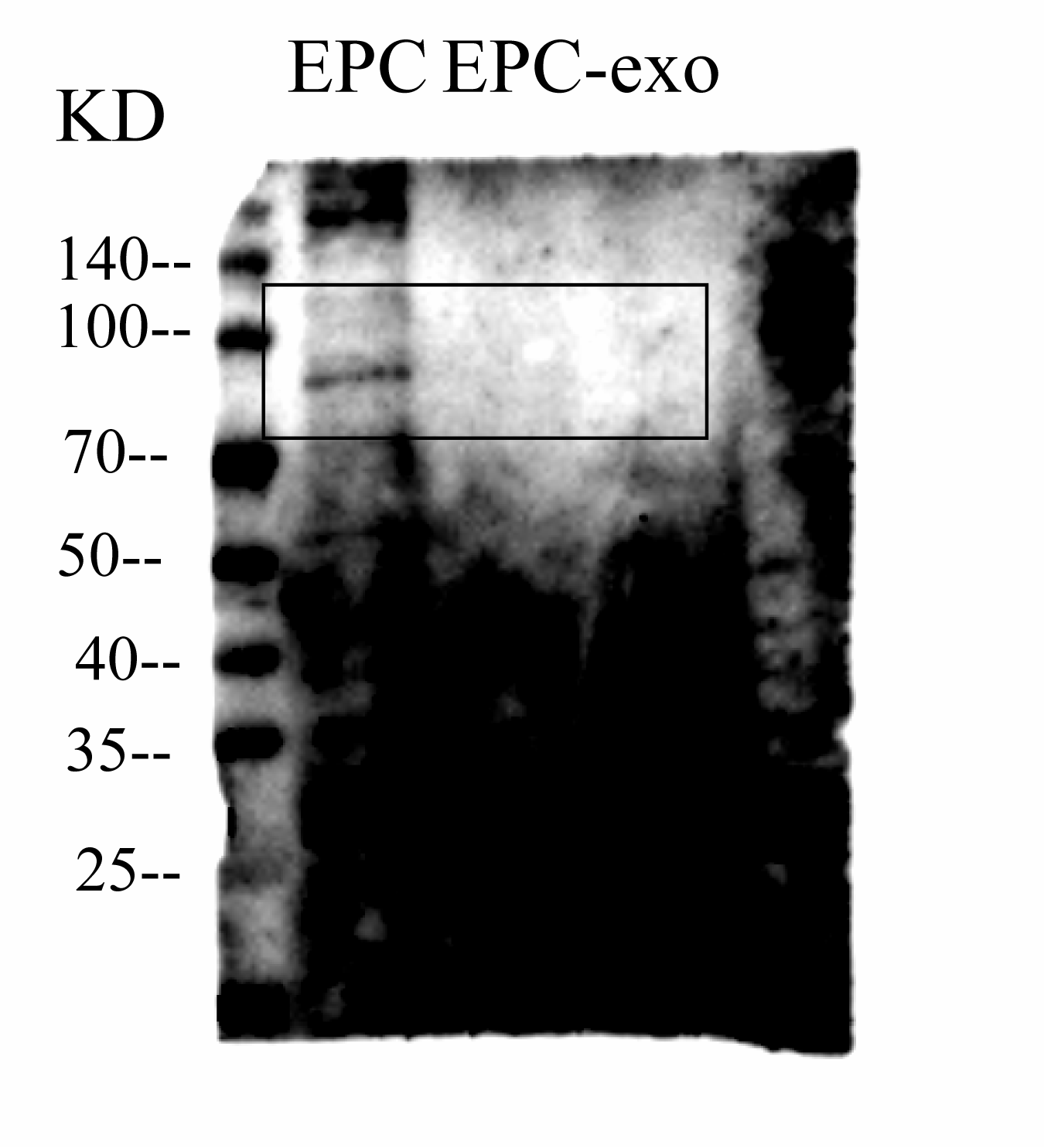


**Figure 2d**


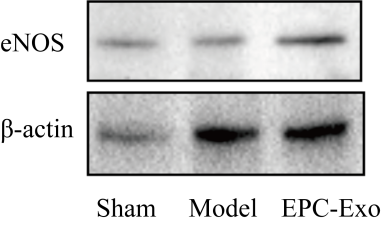
1

2

1 2


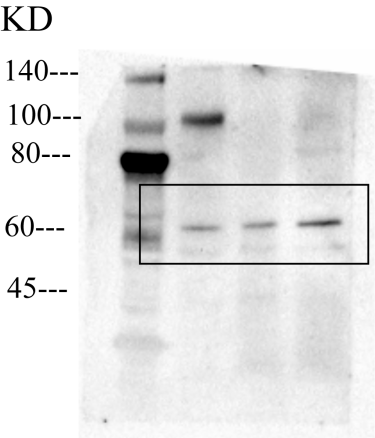

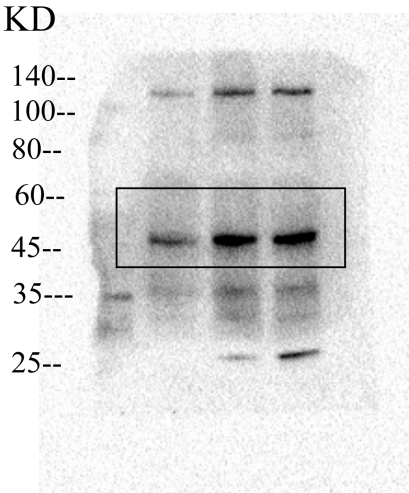


**Figure 3d**


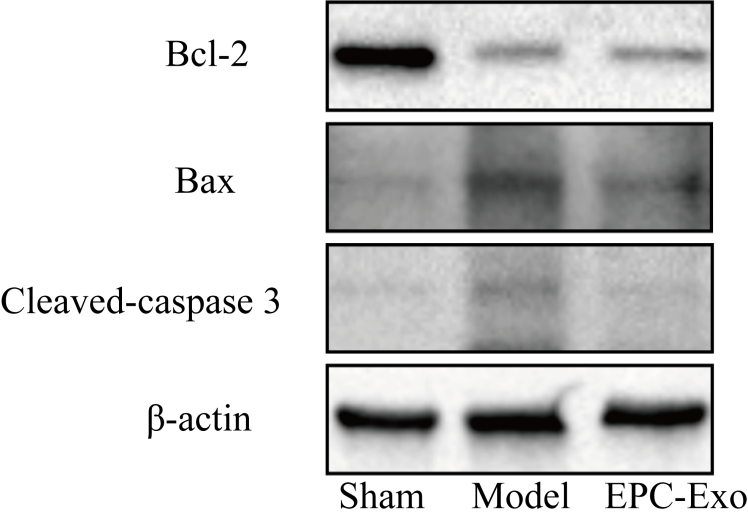
1

2

3

4

1 2


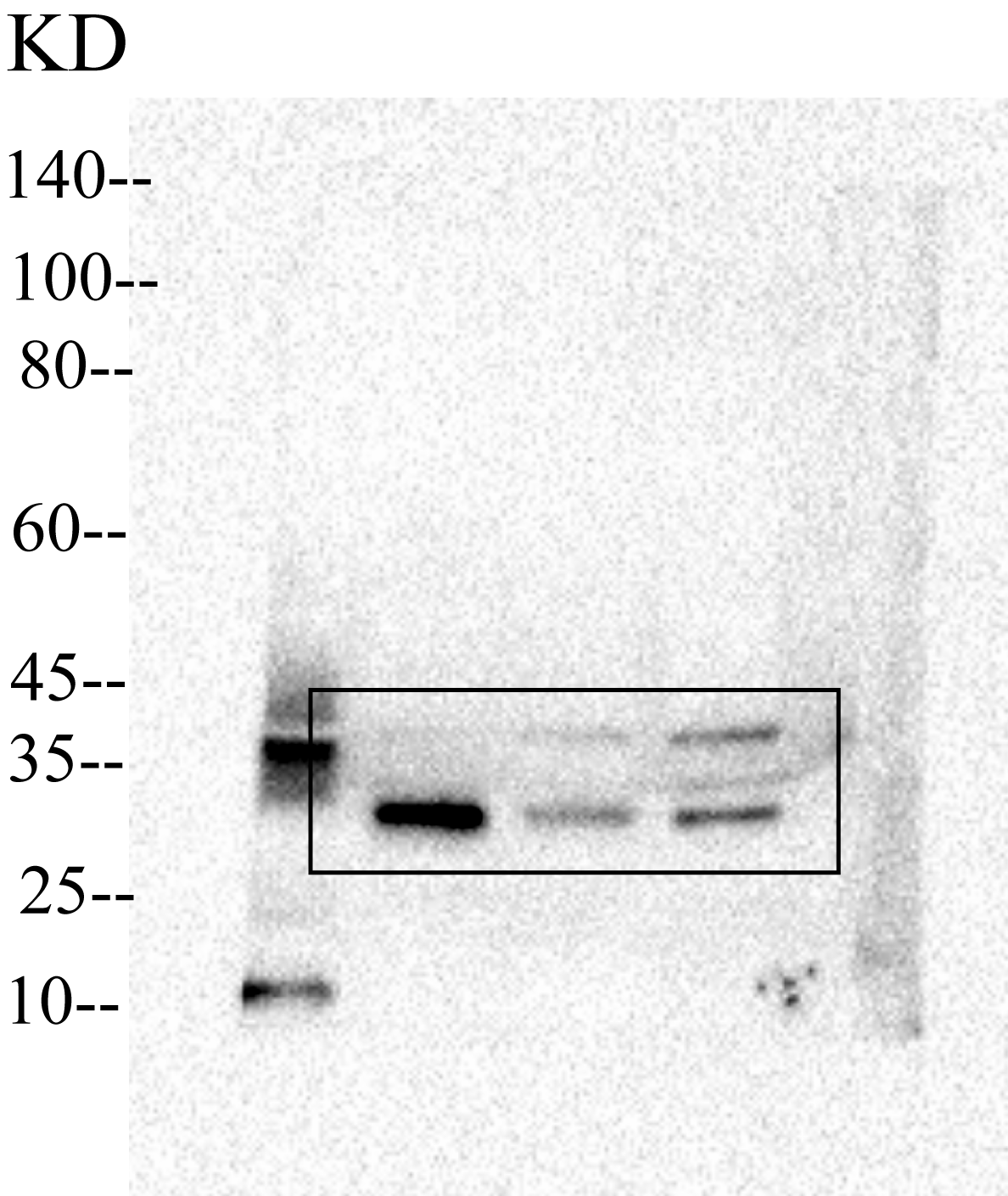

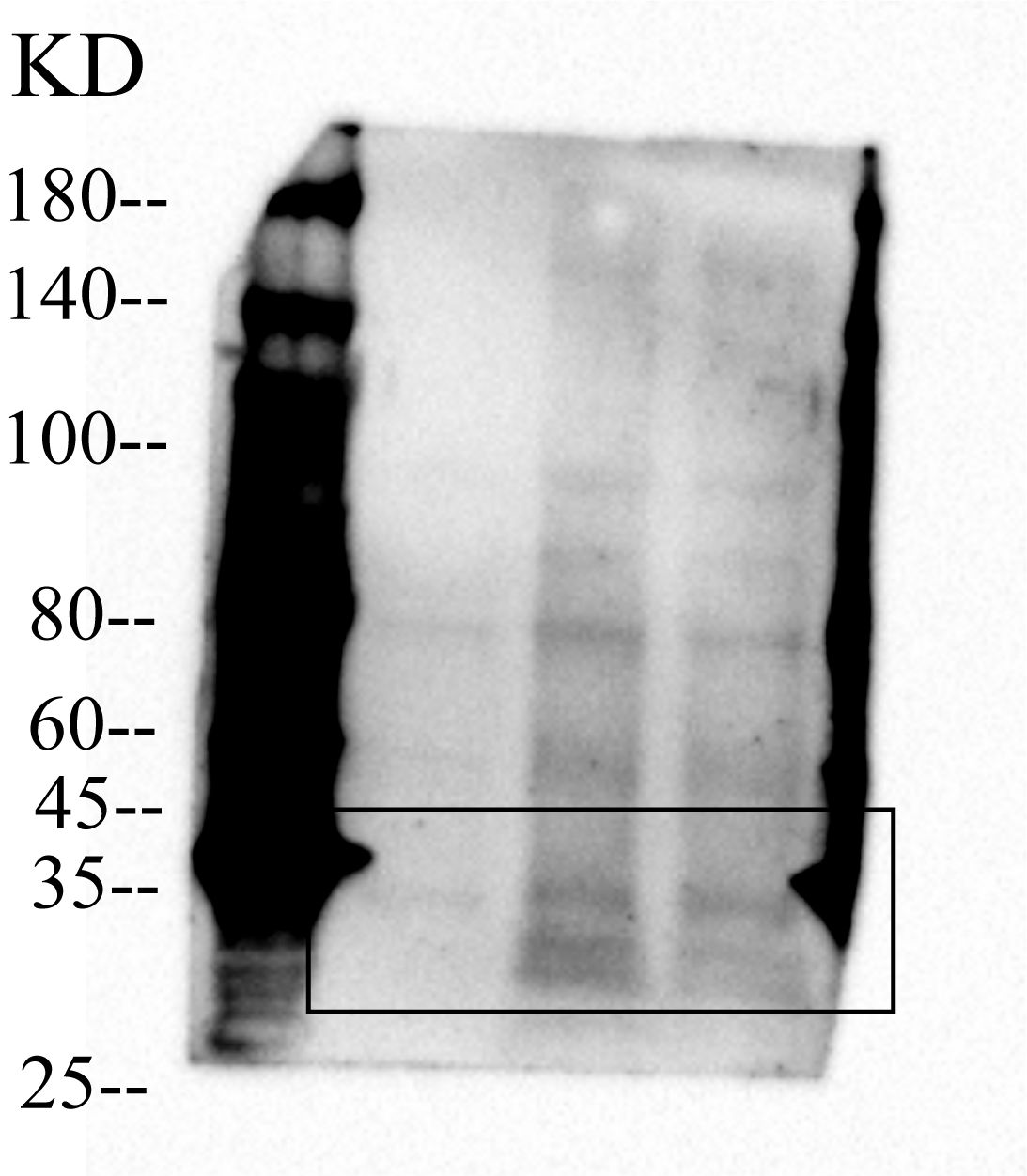


3 4


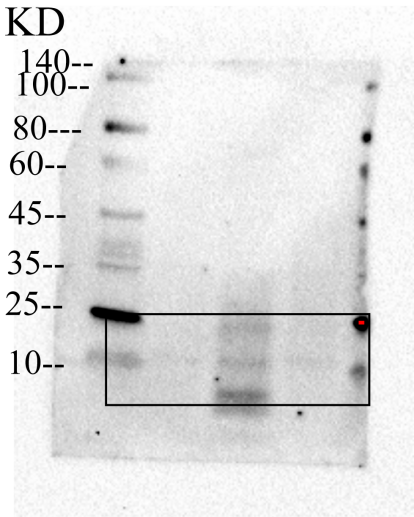

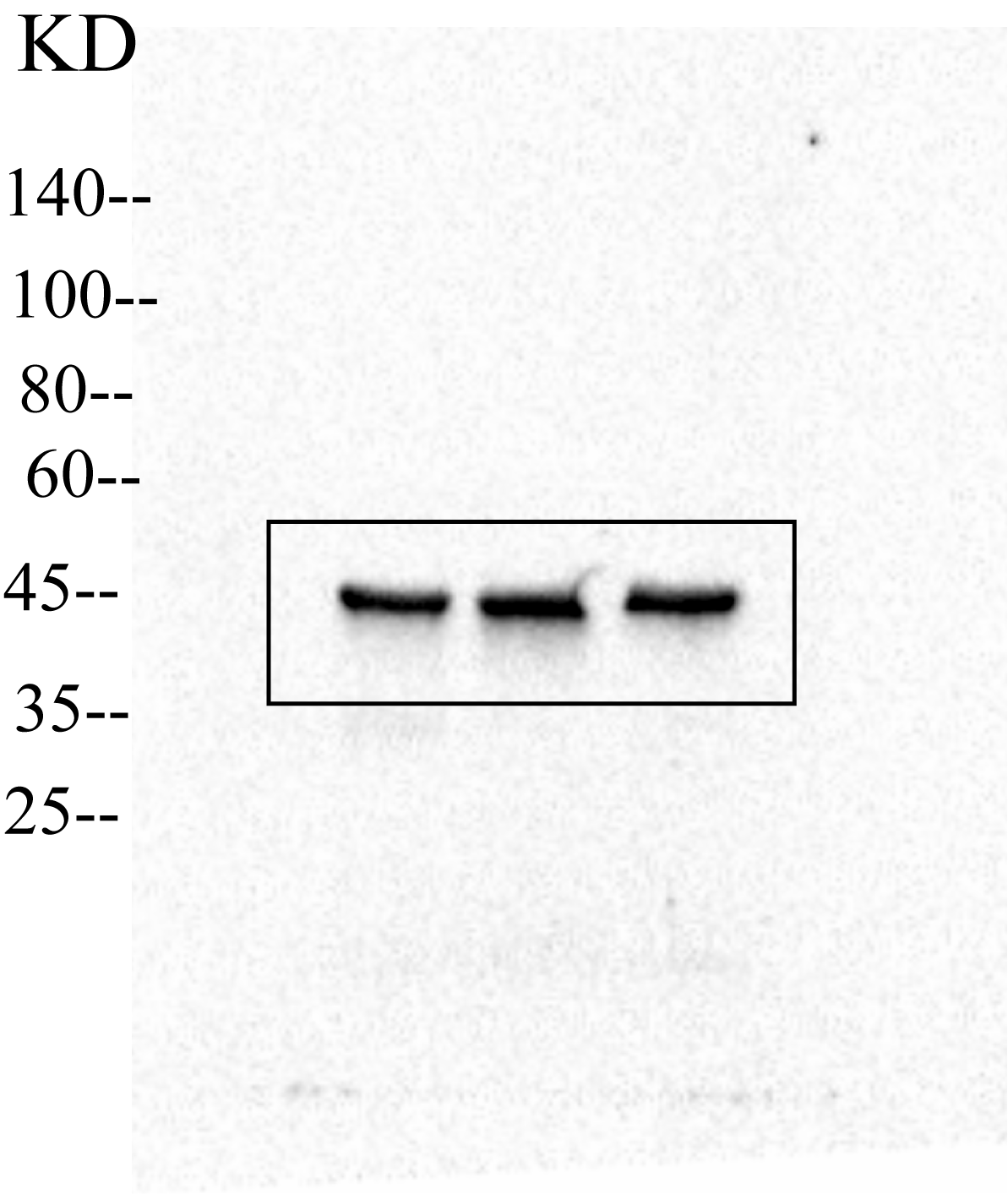


**Figure 5c**


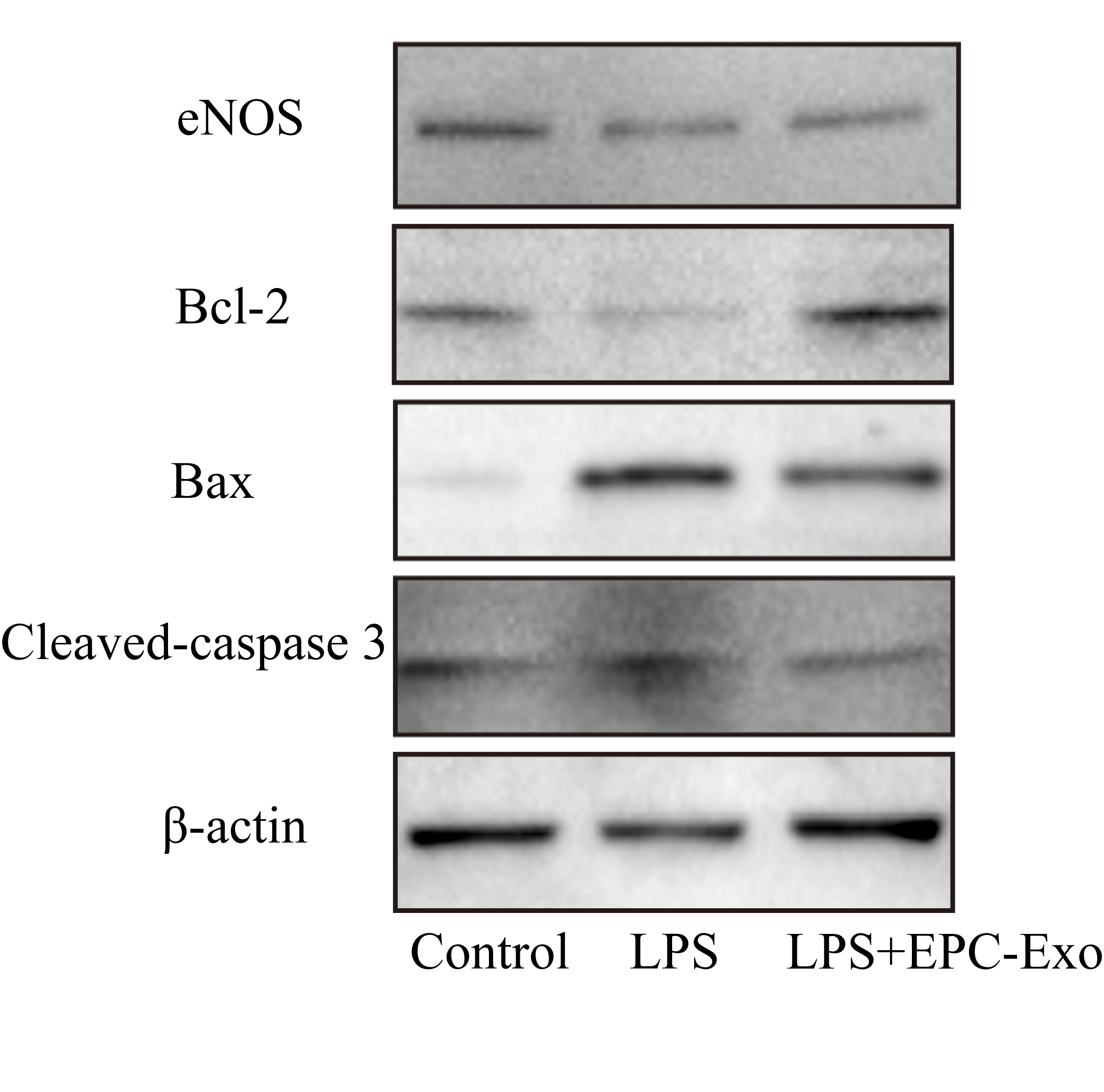


1

2

3

4

5

1 2


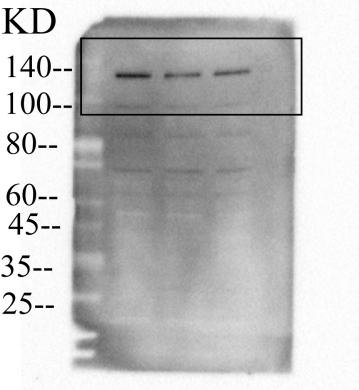


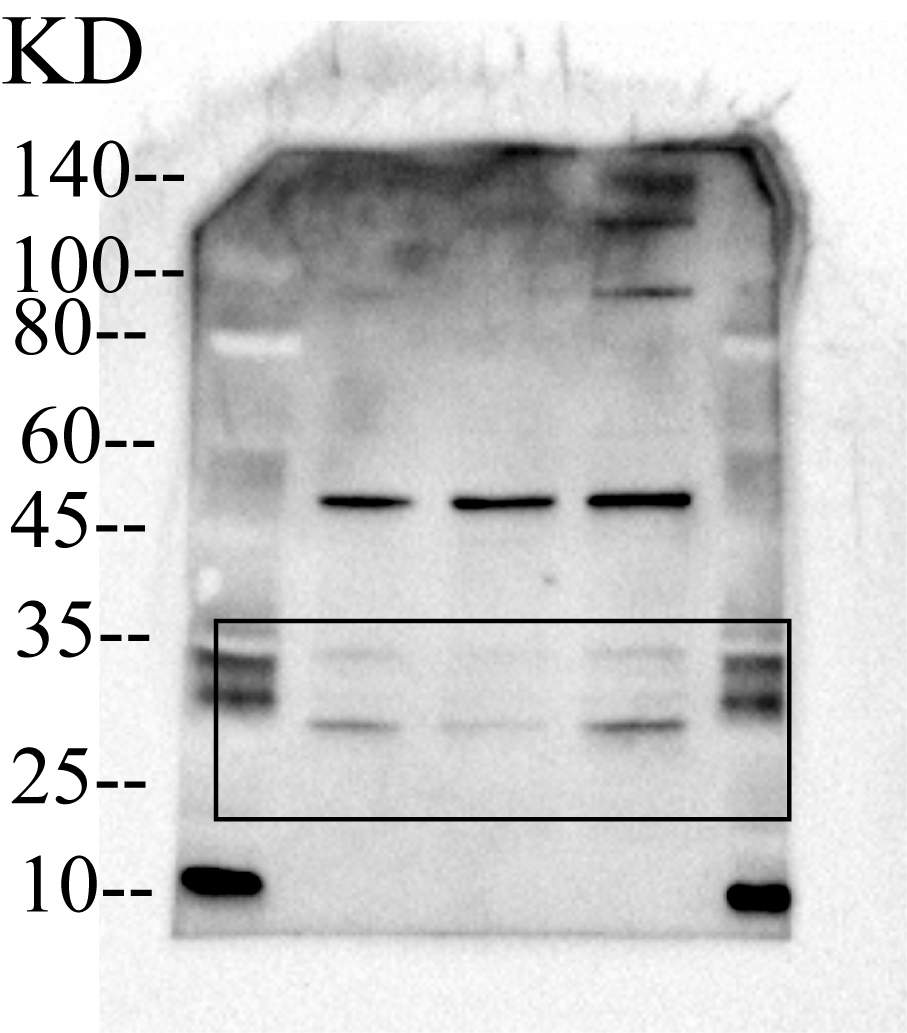


3 4


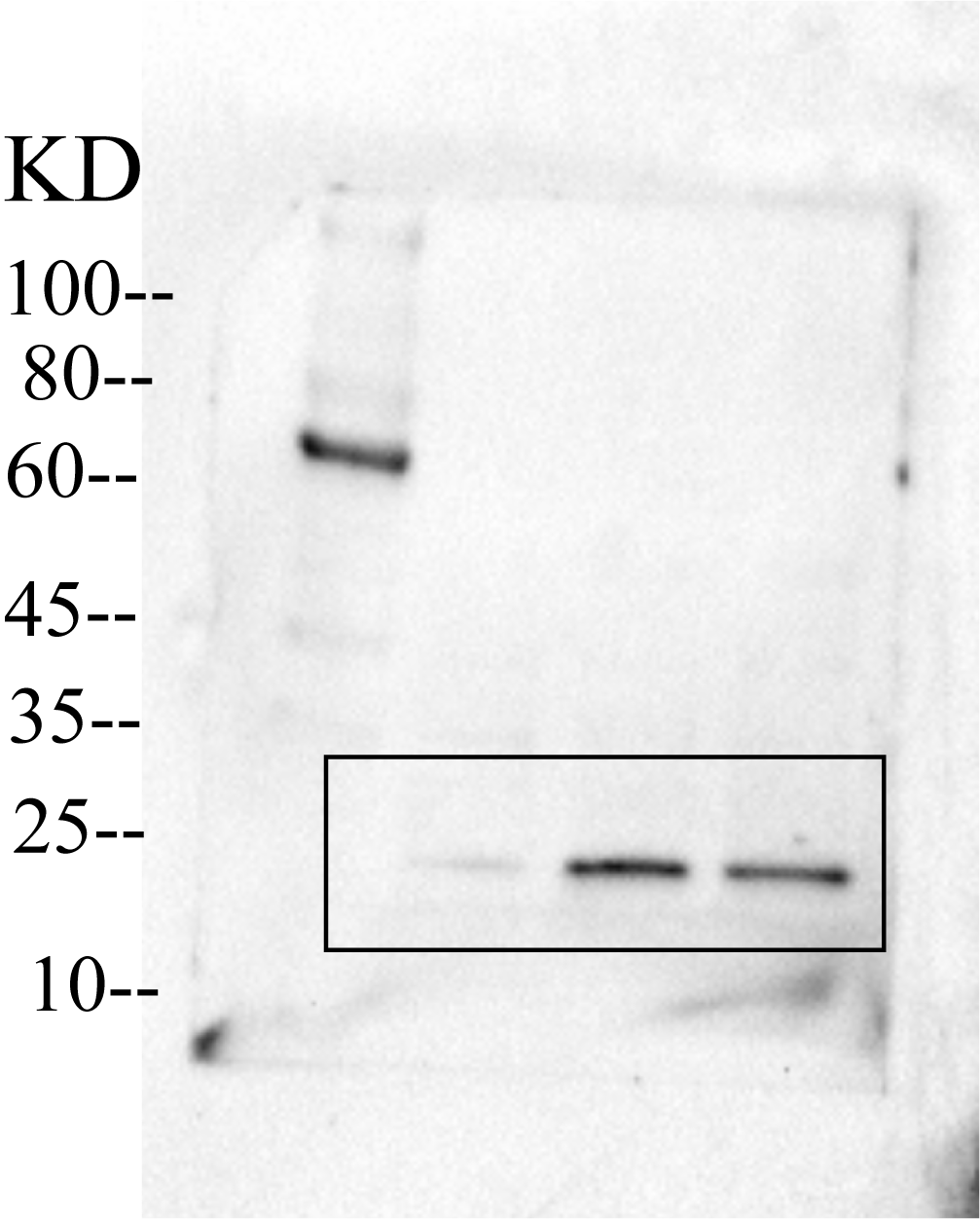

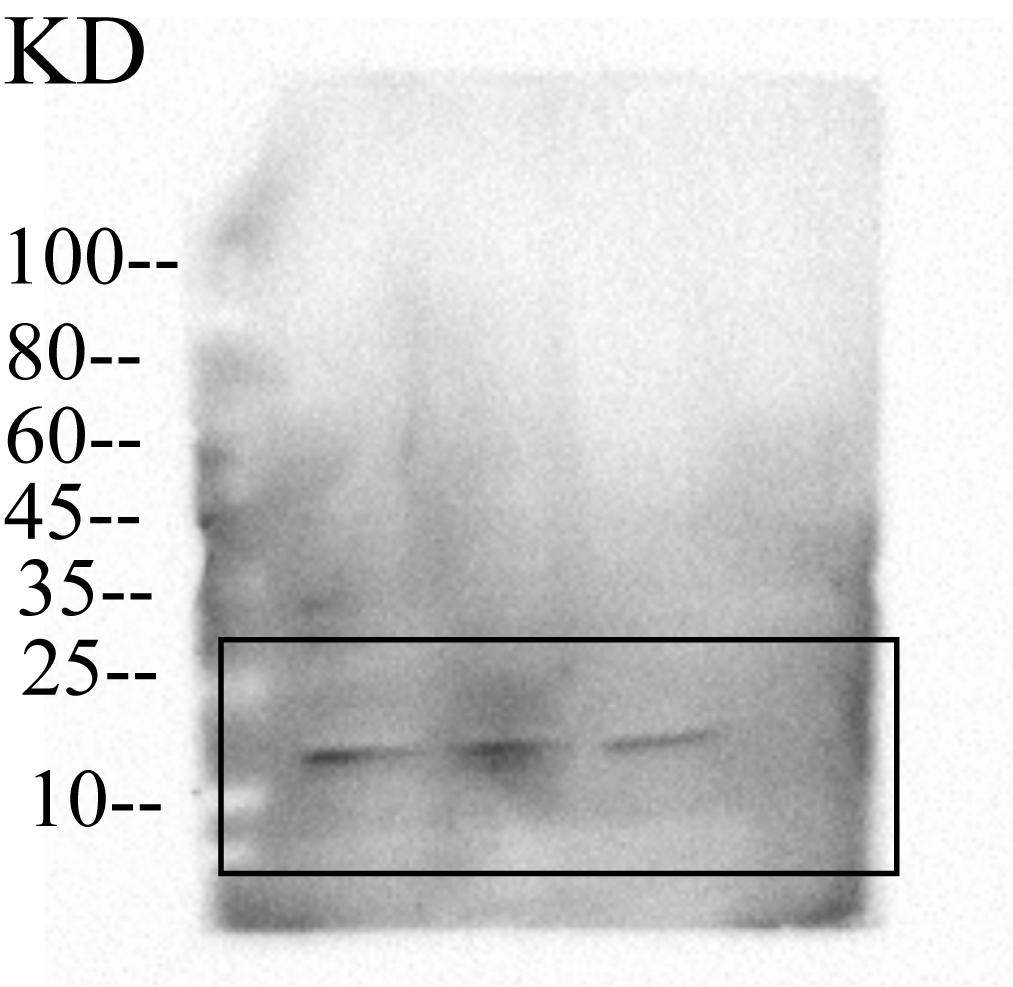


5


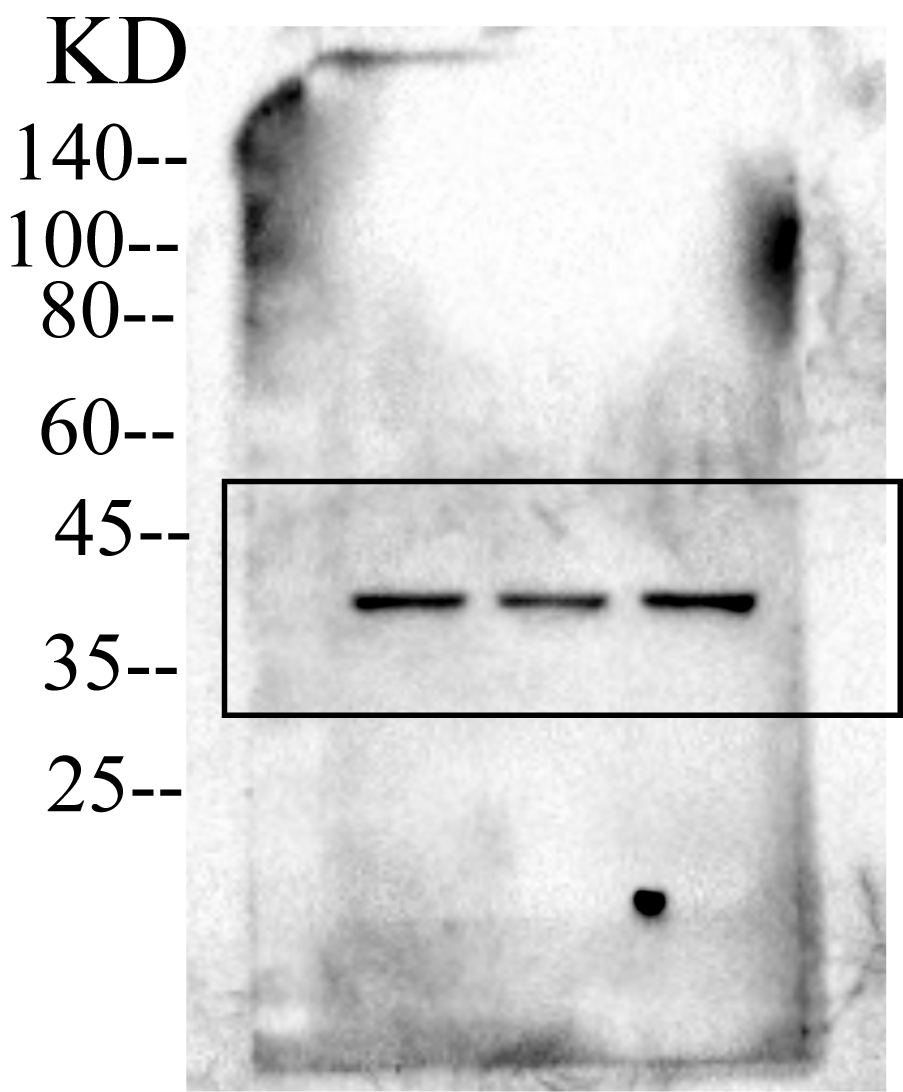

Supplement: Supplementary file 1 — Supplementary Figures. [file 41598_2024_55100_MOESM1_ESM.docx]
